# C3-Alkylation of furfural derivatives by continuous flow homogeneous catalysis

**DOI:** 10.3762/bjoc.19.43

**Published:** 2023-05-03

**Authors:** Grédy Kiala Kinkutu, Catherine Louis, Myriam Roy, Juliette Blanchard, Julie Oble

**Affiliations:** 1 Sorbonne Université, CNRS, Institut Parisien de Chimie Moléculaire, IPCM UMR 8232, F-75005 Paris, France; 2 Sorbonne Université, CNRS, Laboratoire de Réactivité de Surface, LRS UMR 7197, F-75005 Paris, France

**Keywords:** biomass, C–H activation, flow, furfural, homogeneous catalysis

## Abstract

The C3-functionalization of furfural using homogeneous ruthenium catalysts requires the preinstallation of an *ortho*-directing imine group, as well as high temperatures, which did not allow scaling up, at least under batch conditions. In order to design a safer process, we set out to develop a continuous flow process specifically for the C3-alkylation of furfural (Murai reaction). The transposition of a batch process to a continuous flow process is often costly in terms of time and reagents. Therefore, we chose to proceed in two steps: the reaction conditions were first optimized using a laboratory-built pulsed-flow system to save reagents. The optimized conditions in this pulsed-flow mode were then successfully transferred to a continuous flow reactor. In addition, the versatility of this continuous flow device allowed both steps of the reaction to be carried out, namely the formation of the imine directing group and the C3-functionalization with some vinylsilanes and norbonene.

## Introduction

The conversion of biomass derivatives into value-added products is one of the key branches of green chemistry and of the development of a sustainable chemical industry [1–4]. Furfurals, which are versatile platform molecules derived from renewable lignocellulose present in agricultural wastes [5–8], have proven to be of great importance for the preparation of value-added chemicals, biofuels, as well as monomers for materials science [9–15]. In this context, their functionalization is fundamental to further improve their inclusion in fine organic synthesis and industrial processes. For this reason, in recent years, innovative protocols for the formation of new bonds on furfural derivatives have been developed. In particular, their direct functionalization by transition-metal-catalyzed C–H activation processes [16–18] has become a major area of interest where only a few methods have been reported so far. Most examples concern functionalization at C5, which is the most reactive site. In contrast, C3-functionalizations of the formyl-furan unit via directing groups, as well as C4-functionalizations have been much less studied [19–20].

**Scheme 1:**
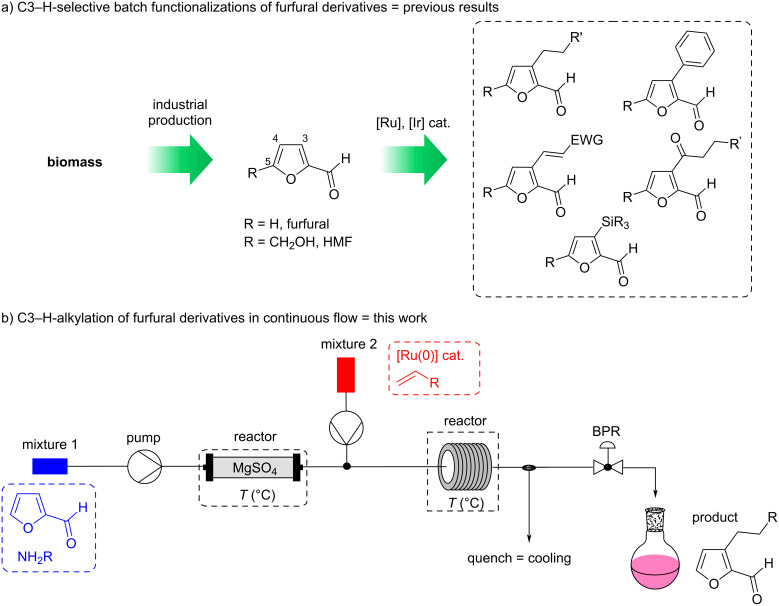
C3-Functionalization of furfural derivatives by C–H activation, a) in batch: previous works, and b) in continuous flow: this work.

Within the framework of a large project oriented towards the selective formation of new bonds from furfural derivatives without changing the redox state of the aldehyde function, we have developed a number of directed Ru(0)-catalyzed C3-functionalizations of furfurylimines, such as alkylation [21], arylation [22], alkenylation [23] and acylation [24], as well as an Ir-catalyzed directed C3-silylation (Scheme 1a) [25]. These batch processes rely on the use of a homogeneous metal catalyst at elevated temperatures necessary to cleave the C3–H bond by oxidative addition. These experimental conditions, easily used in the laboratory, are potentially problematic for scale-up due to efficiency and safety issues (related to the high temperature). Thus, despite the synthetic interest of the molecules that can be obtained, transfers to industry are difficult. In order to circumvent this drawback, we considered transposing these batch reactions to a flow chemistry process.

In recent years, the use of continuous flow chemistry in organic synthesis has increased dramatically and has rapidly become a routine tool for classical synthesis [26–29]. In particular, many efforts have been devoted to the development of flow alternatives for transition-metal-catalyzed cross-couplings [30] and for some C–H functionalizations [31]. Nevertheless, there are very few flow processes that have been implemented to functionalize furfurals, the scarce examples being only based on photochemical processes [32–34]. The current strong interest in continuous flow strategies is related to the modernization of flow equipment providing chemists, not only a unique control of reaction parameters, such as improved mass and heat transfer, but also reduced safety risks and increased reproducibility of the results [29,35–36]. These features should therefore allow us to scale up our directed C3-functionalizations of furfurylimines under safe reaction conditions while providing products in shorter reaction times. In addition, the ability to couple multiple reactors with a flow apparatus could also enable us to perform these functionalizations directly from furfural by forming the imine in a first reactor. It should be noted that, in batch, in-situ imine formation is currently impossible with catalytic or stoichiometric amounts of amine due to decarbonylation of furfural under the reaction conditions [21]. We thus present here an adaptation of our Ru(0)-catalyzed C3-alkylation strategy of furfural derivatives to a continuous flow system (Scheme 1b).

## Results and Discussion

### First optimization with a home-made pulsed-flow setup

**Scheme 2:**
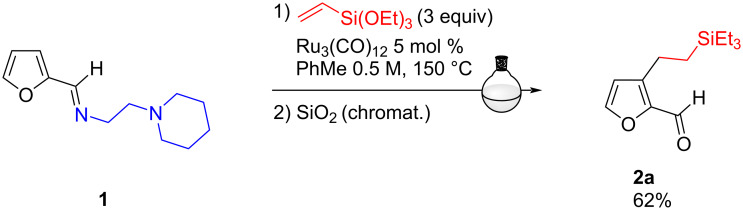
C3-alkylation of bidentate imine **1** performed in batch.

We undertook the optimization of this flow strategy for the C3-alkylation reaction (Murai reaction) [37–38] of the furfurylimine **1** bearing a removable *N*,*N*'-bidentate directing group. In a previous study, this starting material had proved to be the most reactive imine in batch, leading, in the presence of 5 mol % of [Ru_3_(CO)_12_] and 3 equivalents of triethoxyvinylsilane in toluene at 150 °C after 5 h, to the alkylated aldehyde **2a** with 62% yield, after purification on silica gel (Scheme 2) [21,39].

The flow reactions for this first optimization were performed using a home-made setup based on an HPLC apparatus (Jasco) equipped with an injection valve (Rheodyne) comprising a 105 μL loop into which the reaction mixture is loaded and then pushed by the solvent delivered by the HPLC pump (see Supporting Information File 1, p. S8). All the content of the loop is thus sent into the reactor. This system is coupled to a gas chromatography oven, in which the stainless-steel tubular reactor (length: 4.6 m, internal diameter of 0.8 millimeter, corresponding to a volume of 2.31 mL) is placed. The system pressure is controlled by a back-pressure regulator (BPR) to keep a pressure of about 130 bar, i.e., at a pressure much higher than that which causes the solvent (toluene) to boil in the reaction temperature range (150–200 °C). This homemade, pulsed-flow setup was used for optimizing the protocol while saving on reactants and catalyst.

Initial tests with the commercial complex [Ru_3_(CO)_12_] at high temperature with different residence times provided the desired C3-alkylated imine **I2a** in NMR yields ranging from 30% to 65% (Table 1, entries 1–3 and Table S1 in Supporting Information File 1, p. S10). A continuous flow system was thus found to be compatible with the realization of this type of C‒H functionalization. This process led to a significant reduction of the reaction time compared to the batch, in particular by increasing the temperature to 200–250 °C, without significant losses of activity and selectivity. Unfortunately, with this catalyst, repeatability problems were detected (yield fluctuation of approximately 20%) which could be assigned to the low solubility of this catalyst in toluene. In order to overcome these problems, we synthesized triruthenium carbonyl complexes with phosphine ligand(s), namely (triethoxysilyl)ethyl)phosphine **L1** or triphenylphosphine [40–42]. Their synthesis, well-described in the literature, is detailed in Supporting Information File 1 (pp. S3–S6). Moreover, a kinetic study carried out in batch in the presence of the [Ru_3_(CO)_11_(L1)] (**comp1**), [Ru_3_(CO)_10_(L1)_2_] (**comp2**) or [Ru_3_(CO)_9_(L1)_3_] (**comp3**) catalysts allowed to show, on the one hand, the absence of solubility problems, and to discover, on the other hand, that the presence of three **L1** ligands (**comp3**) leads to a reaction rate clearly lower than that of a catalyst carrying one or two ligands (see p. S7 of Supporting Information File 1 for the reaction kinetic curves of catalysts). In addition, the catalyst with a single **L1** ligand (**comp1**) was found to be more reactive than the one with two ligands (**comp2**), and was therefore selected for further optimization. In contrast, comparison of its reaction kinetic curve with that of [Ru_3_(CO)_12_] indicates that **comp1** is slightly less active than [Ru_3_(CO)_12_]. Beside these three catalysts, a fourth one [Ru_3_(CO)_11_(PPh_3_)] **comp4**, was also used for this study.

**Table 1:** Optimization of the catalyst for the alkylation reaction on the homemade pulsed-flow setup.

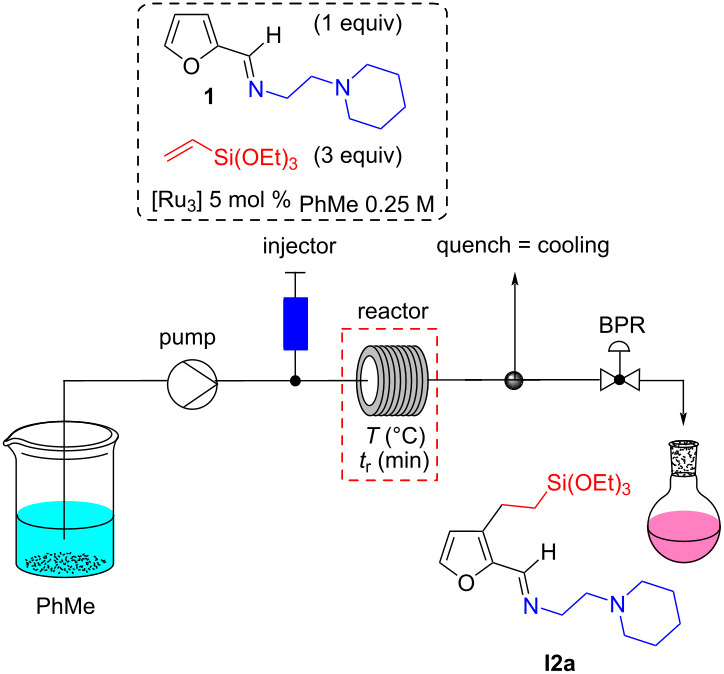					

Entry	[Ru_3_]	*T* [°C]	*t*_r_ [min]	Conv [%]^a^	Yield [%]^a,b^

1	Ru_3_(CO)_12_	165	90	58	45
2	Ru_3_(CO)_12_	200	30	90	65
3	Ru_3_(CO)_12_	250	6	83	63^c^
4	Ru_3_(CO)_11_(L1) **comp1**	200	30	66	56
5	**comp1**	200	46	79	63
6	**comp1**	200	77	95	55

^a^Yields and conversions were calculated by ^1^H NMR using *p*-dinitrobenzene as internal standard; ^b^Non-repeatable results; ^c^repeated four times with four different results ranging from 75% to 53%.

For the continuous flow reaction, we observed, for the same residence time, a slight decrease in performance with **comp1** compared to [Ru_3_(CO)_12_] at 200 °C (Table 1, entries 3 and 4). This can be attributed, as mentioned above, to the slightly faster reaction kinetics of the [Ru_3_(CO)_12_] catalyst compared to that of **comp1**. Nevertheless, the better solubility of **comp1** in toluene allows to get around the problems of reproducibility. Moreover, increasing the residence time to 46 min resulted in 63% NMR yield of **I2a** (Table 1, entry 5), which was very similar to the results obtained with [Ru_3_(CO)_12_] in 30 min. A further increase in residence time to 77 min led to a lower yield (Table 1, entry 6), probably due to products degradation under longer heating.

**Scheme 3:**
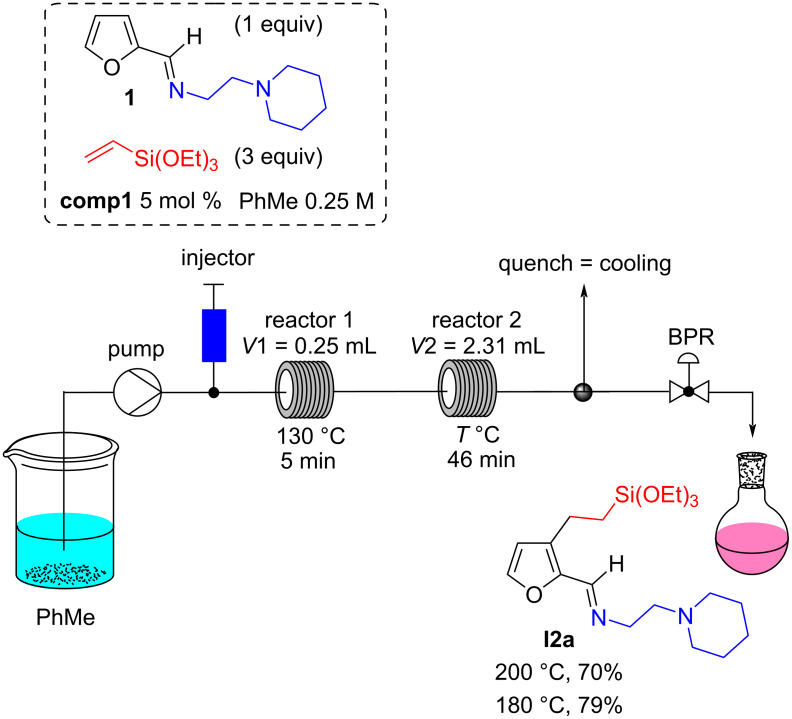
Optimization of the heating for the alkylation reaction on the homemade pulsed-flow setup.

In addition, we found that the **comp1** [Ru_3_(CO)_11_(L1)] was more efficient when the reaction mixture was preheated before being introduced into the reactor at 200 °C. The setup was thus modified (Scheme 3) to include a 0.8-millimeter-diameter stainless-steel preheating loop (outside the oven). An improvement in efficiency was then observed when the reaction mixture was preheated to 130 °C for 5 min. Interestingly, only traces of product **I2a** were observed after 5 min at 130 °C, implying that the Murai reaction was indeed taking place only when passing through the second reactor. Finally, after some optimizations, the temperature in the second reactor could be lowered to 180 °C leading to an NMR yield of C3-alkylated imine **I2a** of 79% (Scheme 3).

**Scheme 4:**
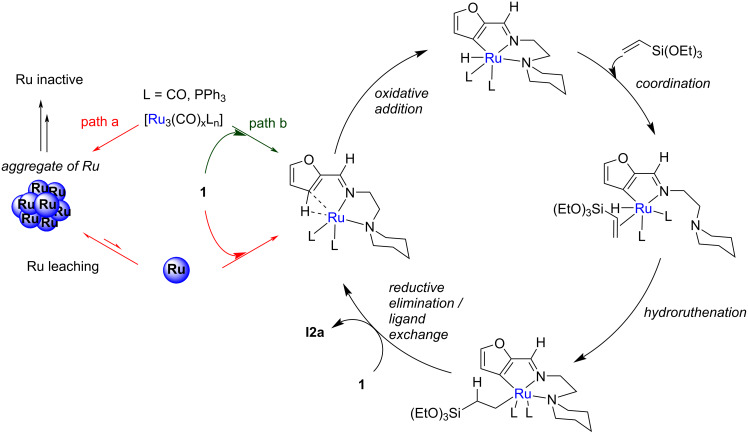
Proposed reaction mechanism for the alkylation reaction with formation of ruthenium aggregates and mononuclear Ru(0) catalyst.

Heat transfer calculations showed us that the reaction mixture rises to the set temperatures in two seconds, and more importantly, that the inlet of reactor 2 is at room temperature after passing through the tube of 50 cm that connects the two reactors. Hence, we rationalized such a performance improvement from a chemical point of view: the [Ru_3_(CO)_12_] complex is known to thermally degrade by deligation, resulting in the formation of ruthenium aggregates [43]. We therefore propose that the active species is a mononuclear carbonyl complex in which the ruthenium is coordinated to the two nitrogen atoms of the directing group (amino-imine). Preheating for 5 minutes at 130 °C would generate it from [Ru_3_(CO)_11_(L)], which would therefore be more accurate to consider as a precatalyst (Scheme 4, path b). The mononuclear complex would then initiate the alkylation reaction at 180 °C following elementary steps previously determined by DFT [21]. Conversely, a high starting temperature would favor the formation of ruthenium aggregates, which could also generate, but less efficiently, the active catalyst (the mononuclear ruthenium(0) species) by leaching (Scheme 4, path a).

**Scheme 5:**
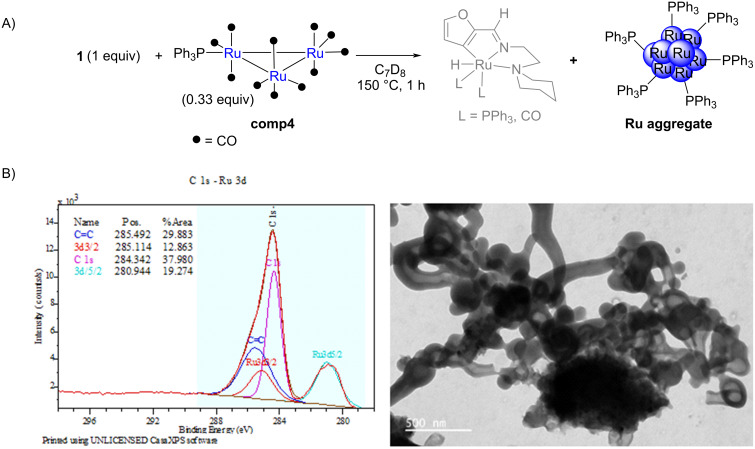
A) Isolation test of a reaction intermediate; B) XPS and TEM (in ethanol) of the recovered solid phase: showing the presence of Ru aggregates.

In order to detect the postulated reaction intermediate (Scheme 4) and the formation of ruthenium aggregates under reaction conditions, imine **1** was treated at 150 °C in toluene for 1 h with 0.33 equiv of **comp4** [Ru_3_(CO)_11_(PPh_3_)], a catalyst analogue to **comp1** but bearing a less expensive phosphine ligand (Scheme 5A). The chosen ratio of imine to catalyst was consistent with the stoichiometric amounts needed to form the postulated intermediate. The temperature of 150 °C was chosen taking into account the efficiency of the batch reaction between imine **1** and triethoxyvinylsilane (3 equiv) in the presence of 5 mol % of this catalyst at 150 °C, which leads in 5 h to the alkylated imine **I2a** with a NMR yield of 77% (conv. 100%).

Even though the reaction intermediate we postulated on Scheme 4 could not be detected, a solid was recovered after evaporation of the solvent and precipitation in pentane. This solid displayed a ^31^P NMR signal at 55.1 ppm (see Supporting Information File 1, p. S24), a value completely different from **comp4** (singlet at 35.06 ppm, see Supporting Information File 1, p. S20), meaning that the ruthenium trimer was no longer present. The TEM analysis of the recovered solid phase (Scheme 5B) showed the formation of large aggregates with high electron density. Moreover, ruthenium was detected by XPS analysis (Scheme 5B); the binding energy of the 3d_5/2_ orbital was 280.94 eV, which corresponds to Ru(0). Double bonds π C=C were also detected in the sample at 285.49 eV, reflecting the presence of the PPh_3_ groups, but no C=O double bonds could be observed (while the presence of a C=O bond was clearly observed on the XPS spectrum of **comp4** (see Supporting Information File 1, p. S6).

**Scheme 6:**
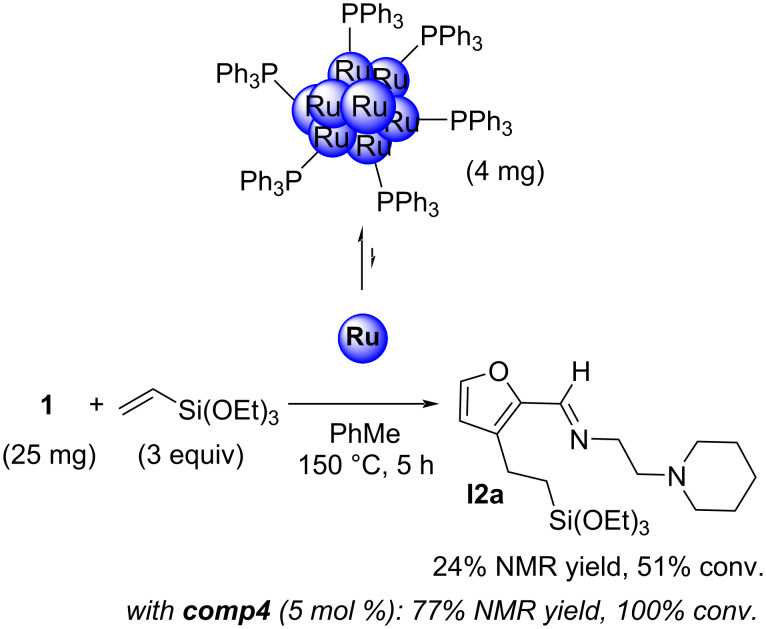
Ruthenium aggregate-catalyzed alkylation reaction.

These Ru aggregates were also used in the reaction with furfurylimine **1** and triethoxyvinylsilane in toluene at 150 °C for 5 h (batch conditions). In this case, only 24% of **I2a** were obtained (Scheme 6), a significant decrease compared to the NMR yield of 77% with **comp4** as (pre)catalyst. These Ru(0) aggregates are therefore active, but the reaction kinetics are slower. While this observation is not a strict confirmation of our hypothesis regarding the formation of a monometallic complex, it is still consistent with it.

### Second optimization with a continuous flow chemistry system

Following these encouraging preliminary results obtained with our home-made, pulsed-flow setup, we decided to run continuous flow experiments using a commercial setup (Vapourtec E-series flow device). This equipment offers the added advantage of being able to couple very simply two reaction steps, and thus to consider the direct functionalization of furfural via the in-situ formation of furfurylimine. Furthermore, as the [Ru_3_(CO)_11_(PPh_3_)] **comp4** catalyst showed an activity and a solubility in batch similar to **comp1**, we continued the optimization with the latter, triphenylphosphine being much cheaper than diphenyl(2-(triethoxysilyl)ethyl)phosphine.

With this setup, the flow system consisted of two mixtures: a mixture A containing furfural (0.7 M) and 2-(piperidin-1-yl)ethane-1,2-diamine (0.7 M) and a mixture B containing vinyltriethoxysilane (1.05 or 2.1 M) and the ruthenium catalyst (1 to 5 mol % with regards to furfural). The flow rates of pumps A and B being equal, the concentration of furfural in mixture A was ≈0.7 M and that of all furfural derivatives was 0.35 M in the final mixture. Mixture A was passed through a fixed bed reactor 1 filled with magnesium sulfate. The residence time depended on the intrinsic volume (*V*i) of this reactor (see Supporting Information File 1, p. S13), and was kept constant at ≈18 min. The mixture B was introduced at the outlet of the fixed bed reactor. A 1 mL stainless steel coil immersed in an oil bath at 130 °C was used as reactor 2, and was installed before the 10 mL reactor 3 (see Supporting Information File 1, pp. S12–S13 for more pictures). By also playing on the flow rate, this allowed us to have conditions close to the best ones observed during optimization on the pulsed-flow device, i.e., *t*_r_2 = 5 min and *t*_r_3 = 50 min (see Table S2 in Supporting Information File 1, p. S11). Product recovery was initiated when the system reached a steady state, based on the dispersion curves provided with the apparatus (see Supporting Information File 1, p. S13). This equipment is a medium pressure system that cannot withstand pressures exceeding 10 bar. Hence, we worked at about 7.5 bar to stay below the boiling curve of toluene in the temperature range used (180–200 °C).

The first experiments performed with this configuration allowed us to validate our hypothesis, namely the possibility of directly functionalizing furfural by forming the imine in situ. Furthermore, the optimized conditions with the pulsed-flow device proved to be effective, as an NMR yield of 62% of the C3-alkylated imine **I2a** was obtained by preheating at 130 °C for 5 min in reactor 1 (after introduction of mixture B), followed by heating at 180 °C for 45 min in reactor 2 (Table 2, entry 1). This allowed us to conclude that pressure does not have an impact on this reaction, since no noticeable difference could be reported when going from ≈130 bar to ≈7.5 bar. The catalytic loading for **comp4** could also be decreased to 1 mol % (Table 2, entries 1, 2, and 4), whereas a diminution in yield was observed when the amount of vinylsilane was decreased to 1.5 equivalents (Table 2, entry 3). The flow alkylation reaction thus appeared to be as efficient with a 1 mol % catalyst loading as with 5 mol %, in contrast to the batch conditions which required 5 mol % of the catalyst. Finally, a very slight improvement was observed by increasing the temperature to 200 °C (entry 5 compared to entry 4 in Table 2).

**Table 2:** Optimization of the alkylation reaction directly from furfural on the continuous flow setup.

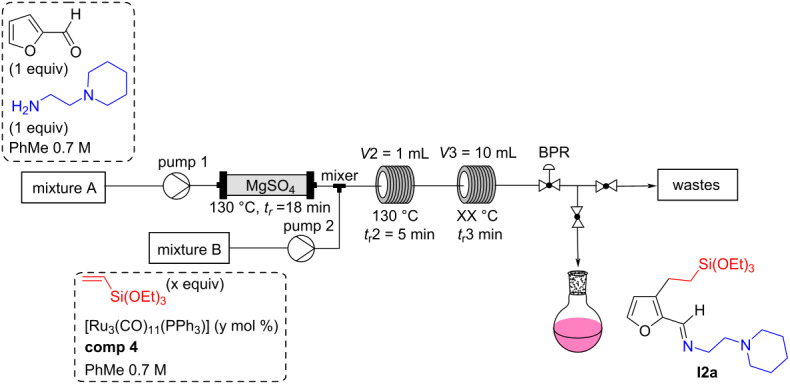					

Entry	**comp4** (y mol %)	Vinylsilane (x equiv)	*t*_r_3 [min]^a^	*T*3 [°C]	Yield [%]^b^

1	5	3	45	180	62
2	2.5	3	50	180	72
3	2.5	1.5	50	180	46
4	1	3	50	180	72
5	1	3	50	200	77
6^c^	1	**3**	50	200	77
7^c^	5	3	45	180	44

^a^A flow rate of 0.22 mL·min^−1^ (0.11 mL·min^−1^ for each pump), allowed us to have a residence time of 45 min in reactor 3, while a flow rate of 0.2 mL·min^−1^ (0.1 mL·min^−1^), provided a residence time of 50 min in reactor 3. ^b^Yields were calculated by ^1^H NMR using *p*-dinitrobenzene as internal standard. ^c^Experiments performed without using the preheating loop in reactor 2.

Finally, it is interesting to note that when the preheating was removed, the same NMR yield was measured with 1 mol % of **comp4** as catalyst (Table 2, entries 5 and 6). On the contrary, when preheating was removed with 5 mol % **comp4** (entry 1 in Table 2), a drastic decrease in yield was observed, from 62% to 44% (entry 1 vs entry 7). This allowed us to assume that such preactivation is no longer necessary with 1 mol % of **comp4**. Thus, a lower catalyst loading, i.e., a lower concentration of the catalyst in the solution, appears to prevent, or at least greatly reduce, the formation of ruthenium aggregates as observed previously, probably by simple dilution effect. As such, the preheating was suppressed for the continuation of our investigations.

### Extending the scope of the C3-alkylation of furfural in continuous flow

**Scheme 7:**
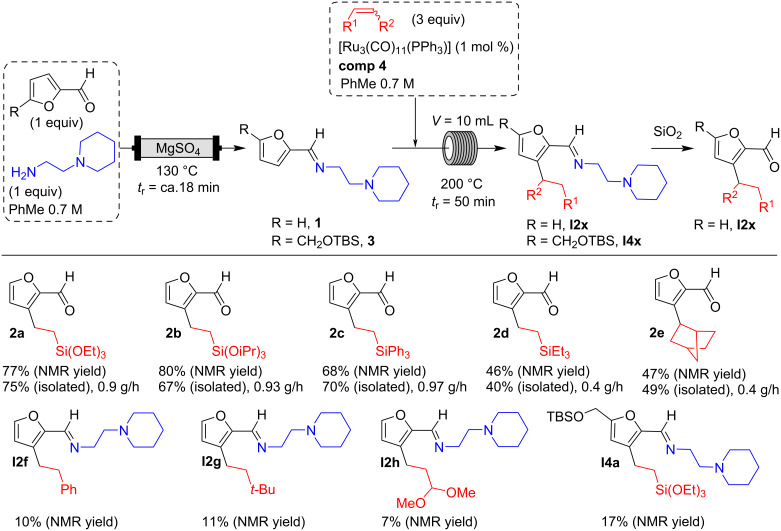
Scope of continuous flow furfural derivative alkylation reaction.

With the optimized conditions in hand (Table 2, entry 6), we were interested in extending the scope of this furfural alkylation reaction using a flow chemistry process to other reactants. For this, after each reaction, an aliquot of the resulting product was recovered for analysis and purification. The NMR yields were calculated on the alkylated imine before purification (based on a starting concentration of furfural of 0.35 M), and the isolated yields corresponded to the C3-alkylated aldehydes after the hydrolysis step that took place during purification. The productivity of each system is given in grams per hour (Scheme 7).

The optimized flow process conditions could be applied to a variety of vinylsilanes: trialkoxy-, triaryl-, and trialkylvinylsilanes, already used in the batch study [21]. The products **2a**–**e** were obtained in good yields and thus with good productivity. Alkenes without silicon in the vinyl position seemed much less reactive, such as a vinylacetal, a hindered olefin (3,3-dimethyl-1-butene), or styrene. In these cases, functionalized furfurals were not isolated. In contrast, norbornene, which has a more reactive double bond due to ring tension, gave *endo* product **2f** with an isolated yield of 49%. On the other side, disubstituted vinylsilanes proved to be ineffective, certainly because of the steric hindrance of the double bond decreasing the kinetics of the hydroruthenation step.

We also wanted to extend this alkylation reaction to a *tert*-butyldimethylsilyl (TBS)-protected 5-HMF derivative. Unfortunately, the yields obtained were very moderate. This reaction having slower kinetics could benefit from being performed at lower temperatures and longer residence time to reduce catalyst degradation. This is unfortunately not possible to implement at the moment with our reactors.

## Conclusion

**Scheme 8:**
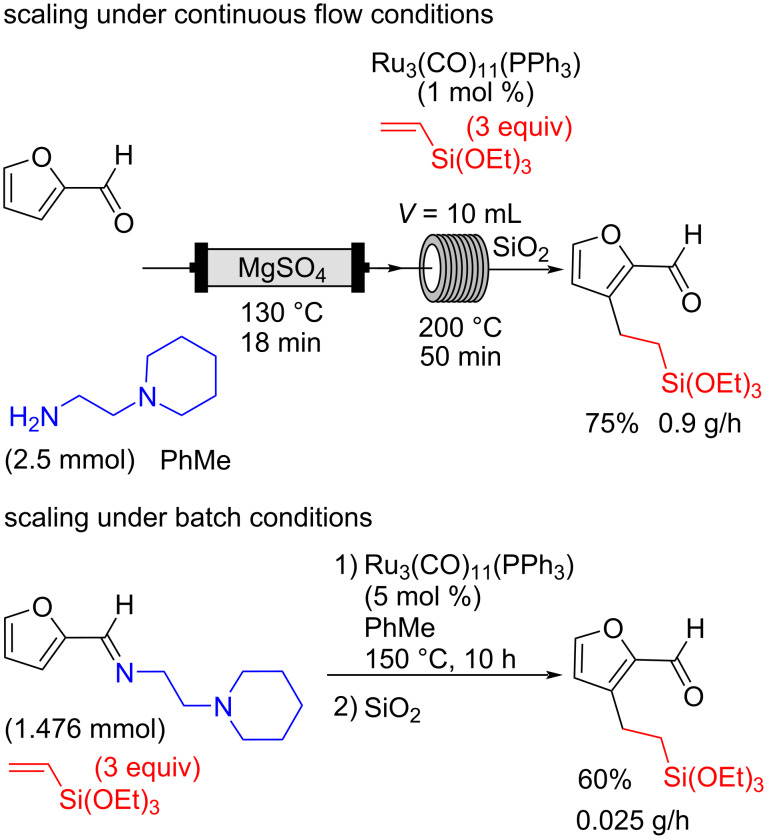
Scaling up comparison: batch and continuous flow conditions.

In conclusion, we have developed a method for the direct 2-step Ru-catalyzed alkylation of the C3–H bond of furfural by flow chemistry, via the preinstallation in a fixed bed reactor of an *ortho*-directing imine group that can be easily removed upon purification on silica. The reaction was found to be very efficient, with a Ru_3_(CO)_11_(PPh_3_) catalyst loading that could be lowered to 1 mol %, allowing for higher yields than batch conditions while requiring 5 times less catalyst. Furthermore, the interest of this flow chemistry approach lays in the scaling up of our reactions. To our great satisfaction, we could show that the productivity of the flow chemistry approach is better than the batch approach with the same catalyst (Scheme 8). This strategy represents a novel method to produce functionalized furfurals, providing synthetically relevant building blocks on a large scale.

## Experimental

### Triphenylphosphine triruthenium undecacarbonyl (**comp4**)

Following a slightly modified procedure compared to the one reported [41], triruthenium dodecacarbonyl (1.4 g, 2.19 mmol, 1 equiv) was dissolved in freshly distilled and degassed THF (0.036 M) at 40 °C. The phosphine ligand (574.40 mg, 2.19 mmol, 1 equiv) dissolved in THF (0.11 M) was then added to the middle. The mixture was stirred at room temperature and treated dropwise with a solution of sodium benzophenone ketyl (about 0.05 equiv added) in THF (0.027 M) via a syringe until the phosphine ligand was completely consumed (monitored by TLC, ≈10 min). The solvent was then evaporated under reduced pressure. The remaining crude was purified by silica gel column chromatography using pentane as eluent, leading to 1.3 g of the desired complex as an orange solid (68% yield). ^1^H NMR (400 MHz, CDCl_3_) δ 7.55–7.37 (m, 15H); ^31^P NMR (162 MHz, CDCl_3_) δ 35.06; XPS BE Ru 3d_5/2_ (281.58). These data are in good agreement with those reported in literature. Crystals were grown from a solution in Et_2_O and identified by X-ray diffraction as a known phase of **comp4** [41].

### General procedure for C3-alkylation of furfural in continuous flow (vapourtec)

*Mixture A*: An oven-dried sealed tube equipped with a magnetic stirrer under argon, was loaded with furfural (240.20 mg, 2.50 mmol, 1 equiv), 2-(piperidin-1-yl)ethanamine (320.55 mg, 2.50 mmol, 1 equiv) and filled with dried toluene to a total volume of 3.5 mL.

*Mixture B*: An oven-dried sealed tube equipped with a magnetic stirrer, was loaded with triphenylphosphine triruthenium undecacarbonyl (1 mol % with regards to furfural) and degassed with argon. Vinyltriethoxysilane (3 equiv with regards to furfural) was then added to the middle, and the mixture was filled with dried toluene to a total volume of 3.5 mL. The mixture was stirred at room temperature to completely dissolve the catalyst.

The solution A is pumped into pump 1 (0.1 mL·min^−1^) and passed through the packed bed reactor which is set at 130 °C containing MgSO_4_. The residence time depends on the intrinsic volume (Vi) of this reactor, and is kept constant at ≈18 min. The solution B is pumped through pump B (0.1 mL·min^−1^). The mixture of the two solutions A and B passed first through the coil reactor at 130 °C and then into a second coil reactor at the desired temperature. Product recovery is initiated when the system reaches a steady state, based on the dispersion curves given by the apparatus. After reaching the steady state an aliquot of the product was taken for ^1^H NMR analysis using *p-*dinitrobenzene as an internal standard.

### 3-(2-(Triethoxysilyl)ethyl)furan-2-carbaldehyde (**2a**)

The reaction of mixture A containing furfural (240.20 mg, 2.50 mmol, 0.7 M) and 2-(piperidin-1-yl)ethanamine (320.55 mg, 2.50 mmol, 0.7 M), with mixture B containing triruthenium undecacarbonyl (22 mg, 0.025 mmol, 0.007 M) and vinyltriethoxysilane (1.43 g, 7.50 mmol, 1.07 M) was conducted by continuous flow chemistry, residence time 1 = 18 min, residence time 2 = 50 min. An aliquot of 0.5 mL of the product mixture was evaporated (93% conv., 77% NMR yield), and the crude was purified by silica gel column chromatography eluting with a mixture of cyclohexane/EtOAc 9:1 to give 38 mg of the desired product as an orange oil (75% yield). ^1^H NMR (300 MHz, CDCl_3_) δ 9.76 (s, 1H), 7.54 (d, *J* = 1.7 Hz, 1H), 6.49 (d, *J* = 1.7 Hz, 1H), 3.81 (q, *J* = 7.0 Hz, 6H), 2.95–2.84 (m, 2H), 1.22 (t, *J* = 7.0 Hz, 9H), 1.01–0.90 (m, 2H). These data are in good agreement with those reported in literature [21].

## Supporting Information

File 1 Experimental and copies of spectra.
