# Supplementary material for: C3-Alkylation of furfural derivatives by continuous flow homogeneous catalysis
Source: Beilstein J Org Chem. 2023 May 3;19:582–92. doi: 10.3762/bjoc.19.43 (PMC10167860; doi:10.3762/bjoc.19.43)

BEILSTEIN JOURNAL OF ORGANIC CHEMISTRY

Supporting Information

for

C3-Alkylation of furfural derivatives by continuous flow homogeneous catalysis

Grédy Kiala Kinkutu, Catherine Louis, Myriam Roy, Juliette Blanchard and Julie Oble

Beilstein J. Org. Chem. **2023,** *19,* 582–592. doi:10.3762/bjoc.19.43

Experimental and copies of spectra

License and Terms: This is a supporting information file under the terms of the Creative Commons Attribution License (https://creativecommons.org/licenses/by/4.0). Please note that the reuse, redistribution and reproduction in particular requires that the author(s) and source are credited and that individual graphics may be subject to special legal provisions.
The license is subject to the *Beilstein Journal of Organic Chemistry* terms and conditions: (https://www.beilstein-journals.org/bjoc/terms)

Table of contents

I.	General information	S2
II.	Preparation of triruthenium complexes	S3
III.	Batch process: kinetic study	S7
IV.	Flow process: optimization	S8
	1. First optimization with an home-made set-up	S8
	2. Second optimization with a continuous flow chemistry system (Vapourtec)	S12
V	Scope of the C3-alkylation of furfural in continuous flow	S14
VI.	Copies of NMR spectra	S16

I. General information

All reactions were performed in oven-dried glassware under argon atmosphere. Reagents and solvents were purchased from commercial sources and generally used as received. CH_2Cl_2 and Et_2O were dried on an MBraun purification system MB SPS-800. THF from the MB SPS-800 was distilled over sodium and benzophenone under nitrogen flow prior to utilization. Acetone was dried over activated 4 Å MS, which was previously flame-dried under vacuum. Commercial anhydrous DMF, toluene, and 1,4-dioxane preserved on molecular sieves were used as is.

Continuous flow chemistry reactions were carried out in a HPLC apparatus JASCO PU980 and a Vapourtec E-series.

Column chromatography was performed on silica gel Merck Geduran® SI 60 (40–63 µm). Analytical thin-layer chromatography (TLC) was performed on Merck 60 F254 silica gel and visualized either with a UV lamp (254 nm or a specific color reagent (potassium permanganate, *p*-anisaldehyde, etc.).

Liquid state NMR spectra (^{1}H, ^{13}C, ^{31}P) were recorded on a Bruker AM 300 MHz or on a Bruker AVANCE 400 MHz spectrophotometer. NMR experiments were carried out at room temperature in $CDCl_3$. Chemical shifts are given in parts per million (ppm) using the $CDCl_3$ residual non-deuterated signals as reference (δ ^{1}H = 7.26 ppm; δ ^{13}C = 77.06 ppm). The terms m, s, d, t, q and br s represent multiplet, singlet, doublet, triplet, quartet, and broad singlet, respectively. Coupling constants (*J*) are given in hertz (Hz). For previously unknown compounds, a combination of ^{13}C, DEPT and 2D experiments (COSY, HSQC, HMBC) were used.

IR spectra were recorded with a Tensor 27 (ATR Diamond) Bruker spectrophotometer. IR spectra were reported as characteristic bands (cm^{-1}). High-resolution mass spectra (ESI–MS or APCI–MS) were acquired using an LTQ-Orbitrap XL from Thermo Scientific (Thermo Fisher Scientific, Courtaboeuf, France) operated in positive ionization mode. Melting points were measured in capillary tubes on a Stuart Scientific SMP3 apparatus and are uncorrected.

XPS analyses were performed using an Omicron Argus X-ray photoelectron spectrometer, equipped with a monochromated Al radiation source (Kα, hν = 1486.6 eV) and a 280 W electron beam power. The emission of photoelectrons from the sample was analyzed at a takeoff angle of 45° under ultra-high vacuum conditions (≤ 10^{-9} mbar). Spectra were carried out with a 100 eV pass energy for the survey scan and 20 eV pass energy for the C 1s, O 1s, N 1s, Cl 2p, Si 2p, B 1s, P 2p, Pd 3d and Ru 3d regions. Element peak intensities were corrected by Scofield factors and the peak areas were determined after subtraction of a linear background. The spectra were fitted using Casa XPS v.2.3.15 software (Casa Software Ltd, U.K.) and applying a gaussian/lorentzian ratio g/l equal to 70/30. [1]

Transmission electron microscopy (TEM) observation of the samples was performed on intact particles that were deposited on copper grids covered with a carbon membrane. TEM images were recorded on a JEOL-JEM2100Plus electron microscope operating at 200 keV (LaB_6 gun).

[1] Fairley, N.; Fernandez, V.; Richard-Plouet, M.; Guillot-Deudon, C.; Walton, J.; Smith, E.; Flahaut, D.; Greiner, M.; Biesinger, M.; Tougaard, S.; Morgan, D.; Baltrusaitis, J. *Appl. Surf. Sci. Adv.* **2021**, *5*, 100112.

The furfurylimine **1** was prepared following our procedure reported in reference [2].

II. Preparation of triruthenium complexes

General procedure for a mono CO-substitution with a phosphine ligand (GP1): following a slightly modified procedure compared to the one reported [3], triruthenium dodecacarbonyl (1 equiv) was dissolved in freshly distilled and degassed THF (0.036 M) at 40 °C. The phosphine ligand (1 equiv) dissolved in THF (0.11 M) was then add to the middle. The mixture was stirred at room temperature and treated dropwise with a solution of sodium benzophenone ketyl (about 0.05 equiv added) in THF (0.027 M) via a syringe until the phosphine ligand was completely consumed (monitored by TLC ≈10 min). The solvent was then evaporated under reduced pressure and the remaining crude was purified by silica gel chromatographic column using pentane as eluent.

General procedure for a di CO-substitution with a phosphine ligand (GP2): Based on a reported procedure [3]. In a two-necked flask under argon and equipped with a condenser, triruthenium dodecacarbonyl (1 equiv) and trimethylamine *N*-oxide dihydrate (0.08 equiv) were suspended in heptane (0.013 M). The phosphine ligand, diluted in minimal heptane if solid, was then added to the medium. The reaction mixture was heated at reflux for 16 min, after cooling to room temperature, the solvent was evaporated under reduced pressure and the remaining crude was purified by silica gel column chromatography using a mixture of pentane/Et_2O 8:2 as eluent.

(Diphenyl(2-(triethoxysilyl)ethyl)phosphine)triruthenium undecacarbonyl (Comp1)

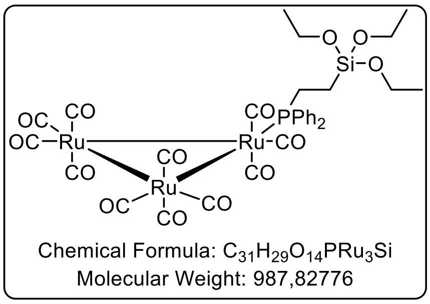

Prepared according to **GP1**: the reaction of triruthenium dodecacarbonyl (200 mg, 0.312 mmol) with diphenyl(2-(triethoxysilyl)ethyl)phosphine (117.44 mg, 0.312 mmol) in THF (12 mL) gave 149 mg of the desired product as an orange solid (48% yield). **^{1}H NMR** (400 MHz, $CDCl_3$) δ 7.52 – 7.41 (m, 10H), 3.73 (q, *J* = 7.0 Hz, 6H), 2.58 – 2.45 (m, 2H), 1.17 (t, *J* = 7.0 Hz, 9H), 0.53 – 0.41 (m, 2H). **^{31}P NMR** (162 MHz, $CDCl_3$) δ 33.13. These data are in good agreement with those reported in literature. [3]

Crystallographic data and structure refinement for **comp1**
A suitable crystal was selected, mounted onto a cryoloop and transferred into a cold nitrogen gas stream. Intensity data were collected with a Bruker Kappa APEX-II CCD diffractometer using a graphite-monochromated MoKα radiation (λ = 0.71073 Å). Data collection was performed at 200 K with the Bruker APEXIII suite. Unit-cell parameters determination, integration and data reduction were carried out with SAINT program. SADABS was used for scaling and absorption corrections. [4] The structure was solved with SHELXT [5] and refined by full-matrix least-squares methods with SHELXL using Olex2 software package [6] All non-hydrogen atoms were refined anisotropically. This structure was

[2] Pezzetta, C.; Veiros, L. F.; Oble, J.; Poli, G. *Chem. Eur. J.* **2017**, *23*, 8385.
[3] Carniato, F; Secco, A.; Gatti, G.; Marchese, L.; Sappa, E. *J Sol-Gel Sci Technol* **2009**, *52*, 235.
[4] Palatinus, L.; Chapuis, G. *Journal of Applied Crystallography* **2007**, *40*, 786.
[5] Sheldrick, G. M. *Acta Crystallographica Section C* **2015**, *71*, 3.
[6] Dolomanov, O.V.; Bourhis, L.J.; Gildea, R.J.; Howard, J.A.K.; Puschmann, H. *Journal of Applied Crystallography* **2009**, *42*, 339.

deposited at the Cambridge Crystallographic Data Centre with number CCDC 2240638 and can be obtained free of charge via www.ccdc.cam.ac.uk.

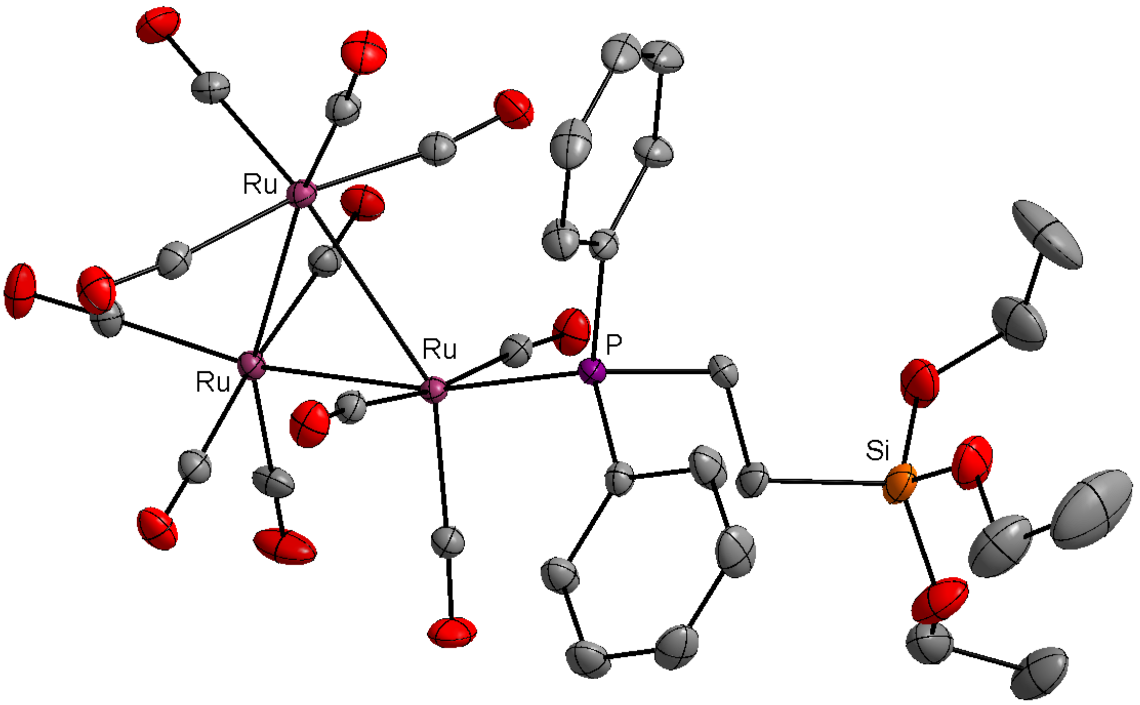

Figure S1: Crystal structure representation of comp1. Ellipsoids are shown with 30% probability. Two molecules of interest are present in the asymmetric unit (space group $P2_1/n$) but only one is depicted for sake of clarity. All hydrogen atoms and the secondary position (occ. 0.5) of one disordered ethoxy group are not shown for simplicity. Red: oxygen atoms. Grey: carbon atoms.

Crystallographic data for comp1

	**1**
CCDC deposit number	*2240638*
**Empirical formula**[a]	$C_{31}H_{29}O_{14}PRu_3Si$
**Moiety Formula**	$C_{31}H_{29}O_{14}PRu_3Si$
**Formula weight (g/mol)**	987.81
**Temperature (K)**	200
**Crystal system**	Monoclinic
**Space group**	$P2_1/n$
**a (Å)**	11.2156(5)
**b (Å)**	20.3951(9)
**c (Å)**	32.9753(14)
**α (°)**	90
**β (°)**	94.088(2)
**γ (°)**	90
**Volume (Å^3)**	7523.7(6)
**Z**	8
**ρ_{calc} (g/cm^3)**	1.744
**Absorption coefficient μ (mm^{-1})**	1.326 (MoKα)
**F(000)**	3904.0

**Crystal size (mm²)**	0.21 × 0.17 × 0.05
**Wavelength λ (Å)**	1.54178
**2Θ range (°)**	2.476 - 61.6
**Miller indexes ranges**	-16 ≤ h ≤ 15, -29 ≤ k ≤ 29, -41 ≤ l ≤ 47
**Measured reflections**	142649
**Unique reflections**	23422
**R_{int} / R_{sigma}**	0.0494 / 0.0418
**Reflections [I ≥ 2σ(I)]**	15606
**Restraints**	18
**Parameters**	946
**Goodness-of-fit F^2**	1.038
**Final R indexes[b c] [all data]**	R_1 = 0.0789, wR_2 = 0.0693
**Final R indexes[b c] [I ≥ 2σ(I)]**	R_1 = 0.0393, wR_2 = 0.1400
**Largest diff. peak/hole (e/Å³)**	0.99/ -0.72
**Flack parameter**	Not applicable

[a] Including solvent molecules (if presence)

[b] $R1 = \sum||F_o| - |F_c|| / \sum|F_o|$ [c] $wR2 = \sqrt{\sum(w(F_o^2 - F_c^2))/\sum(w(F_o^2)^2)}$

Bis(diphenyl(2-(triethoxysilyl)ethyl)phosphine)triruthenium decacarbonyl (comp2)

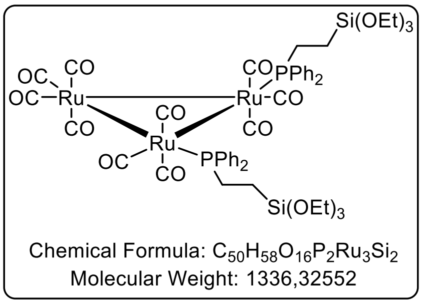

Prepared according to **GP2**: the reaction of triruthenium dodecacarbonyl (50 mg, 0.078 mmol) with diphenyl(2-(triethoxysilyl)ethyl)phosphine (64.60 mg, 0.172 mmol) in heptane (6 mL) gave 65.6 mg of the desired product as an deep orange solid (63% yield). **^{1}H NMR** (300 MHz, $CDCl_3$) δ 7.53 – 7.34 (m, 20H), 3.73 (q, *J* = 7.0 Hz, 12H), 2.56 – 2.42 (m, 4H), 1.17 (t, *J* = 7.0 Hz, 18H), 0.56 – 0.41 (m, 4H). **^{31}P NMR** (122 MHz, $CDCl_3$) δ 33.35, 31.90. These data are in good agreement with those reported in literature. [3]

Tris(diphenyl(2-(triethoxysilyl)ethyl)phosphane)triruthenium nonacarbonyl (comp3)

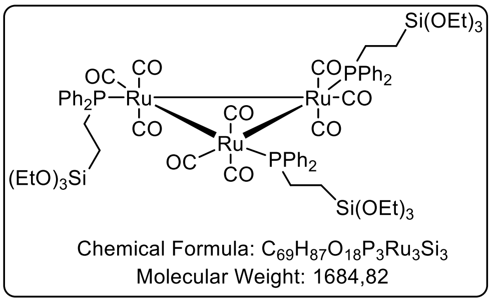

Following a reported procedure, [7] in a round-bottomed flask equipped with a condenser under argon atmosphere, triruthenium dodecacarbonyl (497 mg, 0.78 mmol) and diphenyl(2-(triethoxysilyl)ethyl)phosphine (940 mg, 2.50 mmol) were dissolved in hexane (63 mL). The mixture was stirred at 60 °C overnight and then cooled to room temperature. The solvent was evaporated under reduced pressure and the oily residue was triturated in a 9:1 methanol/water mixture to give 1.3 g (quantitative yield) the desired product as a deep red solid. **^{1}H NMR** (400 MHz, $CDCl_3$) δ 7.51 – 7.29 (m, 30H), 3.73 (q, *J* = 7.0 Hz, 18H), 2.58 – 2.46 (m, 6H), 1.17 (t, *J* =

[7] Dallmann, K.; Buffon, R. *J. Mol. Catal. Chem. A* **2002**, *185*, 187.

7.0 Hz, 27H), 0.58 – 0.43 (m, 6H). ^{13}C NMR (101 MHz, $CDCl_3$) δ 136.7, 136.2, 132.5, 132.4, 129.3, 128.1, 128.0, 58.4, 26.8, 18.2, 4.1. ^{31}P NMR (162 MHz, $CDCl_3$) δ 34.70. **XPS BE** Ru $3d_{5/2}$ (281.01).

XPS for **comp3** :

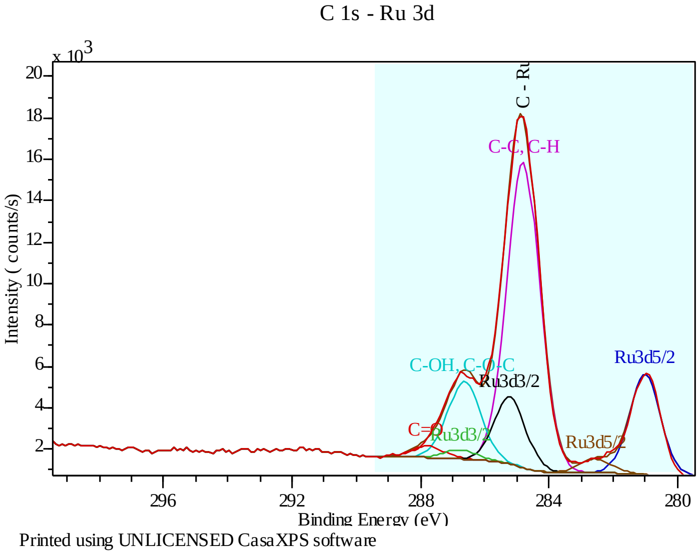

Triphenylphosphine triruthenium undecacarbonyl (comp4)

Prepared according to **GP1**: the reaction triruthenium dodecacarbonyl (1.4 g, 2.19 mmol) with triphenylphosphine (574.40 mg, 2.19 mmol) in THF gave 1.3 g of the desired product as an orange solid (68% yield). **^{1}H NMR** (400 MHz, $CDCl_3$) δ 7.55 – 7.37 (m, 15H). **^{31}P NMR** (162 MHz, $CDCl_3$) δ 35.06. **XPS BE** Ru $3d_{5/2}$ (281.58). These data are in good agreement with those reported in literature. [8] Crystals were grown from a solution in Et_2O and identified by X-ray diffraction as a known phase of **comp4**. [8]

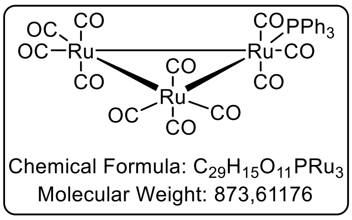

XPS for **comp4** :

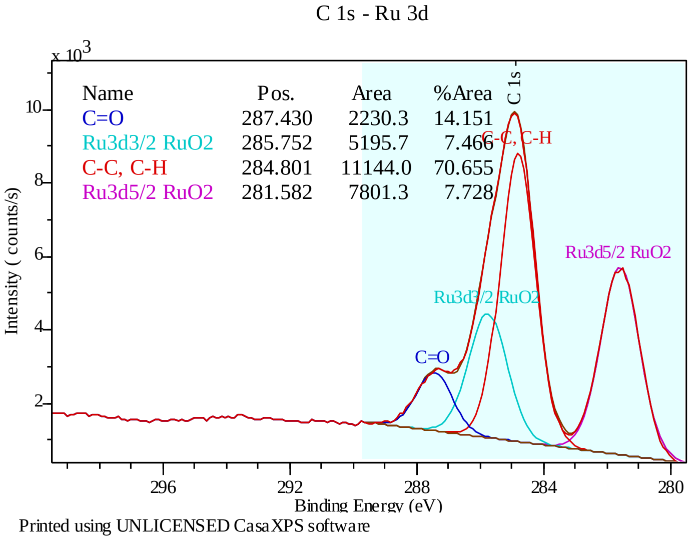

[8] Forbes, E. J.; Goodhand, N.; Jones, D. L.; Hamor, T. A. *J. Organomet. Chem.* **1979**, *182*, 143–154.

III. Batch process: kinetic study

A sealed tube equipped with a magnetic stirrer was charged with a ruthenium catalyst (5 mol %). The tube was degassed three times with argon and a solution of the imine **1** (1 equiv) in toluene (0.5 M) was added followed by the vinyl silane partner (3 equiv). The tube was sealed and heated at 150 °C for the appropriate amount of time, and the mixture is directly cooled by being immersed in an acetone/ice bath to stop the reaction. Then a solution of dinitrobenzene in toluene was added to the mixture before filtration through a Celite pad and washed with CH_2Cl_2. The solvent was removed at reduced pressure and the crude product was analyzed by ^1H NMR.

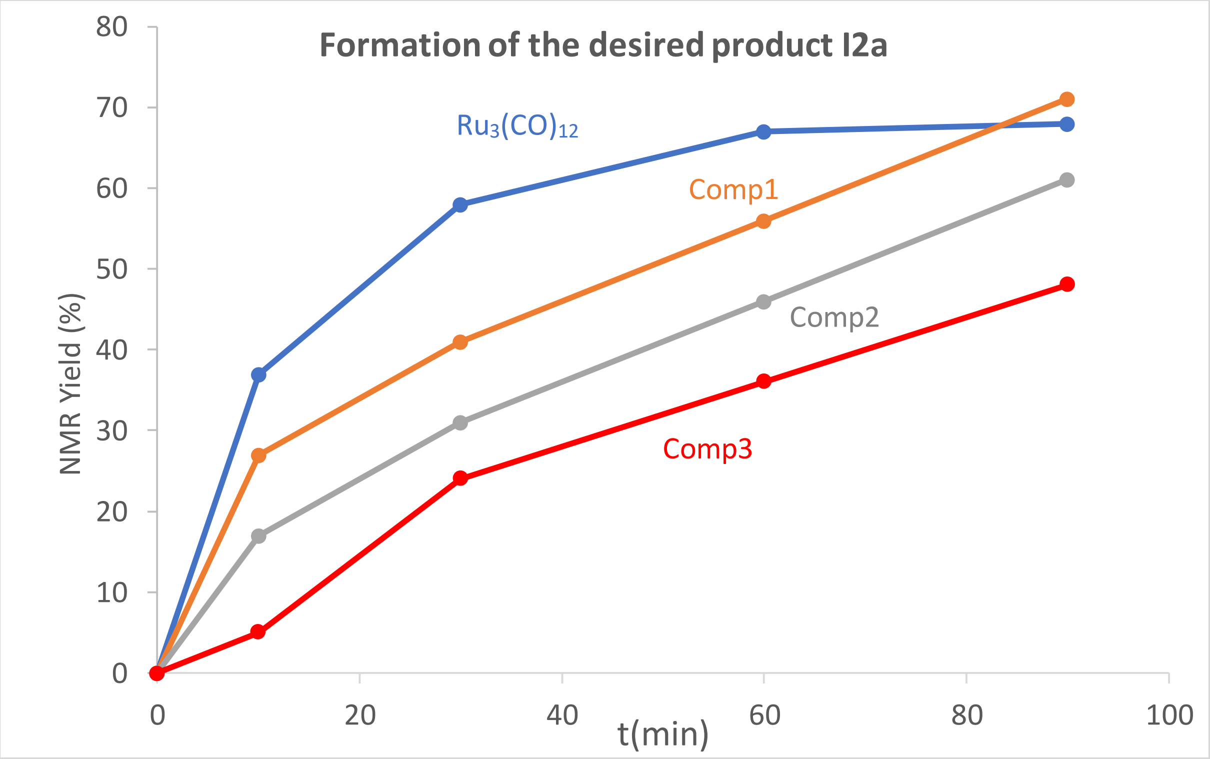

IV. Flow process: optimization

1. First optimization with a home-made setup

Home-made setup including:

- A HPLC apparatus (Jasco) equipped with an injection valve (Rheodyne) comprising a 105 µL loop
- A gas chromatography oven, in which the stainless steel tubular reactor (length: 4.6 m, volume 2.31 mL) with an internal diameter of 0.8 mm is placed.
- A back-pressure regulator (BPR) to keep a pressure of about 130 bar

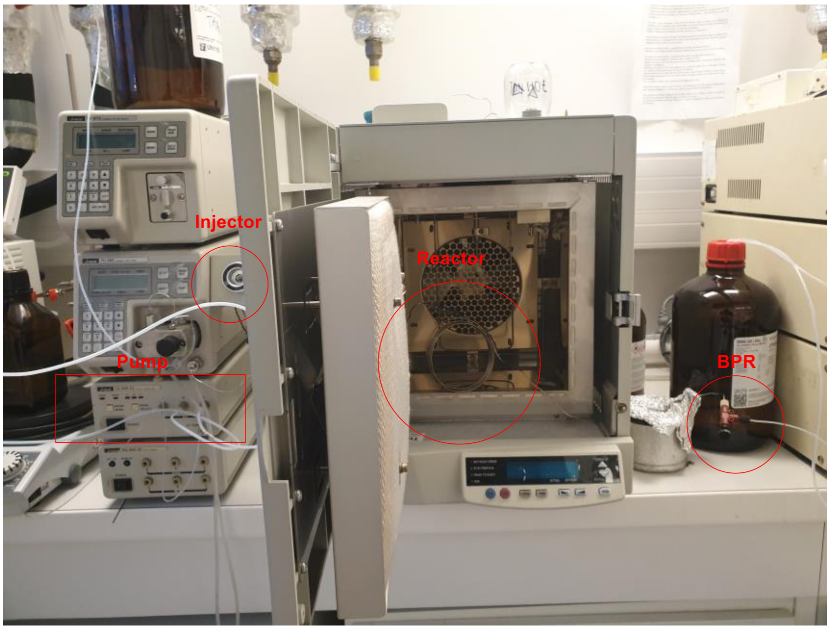

General procedure (GP3): An oven-dried round-bottomed flask equipped with a magnetic stirrer was charged with the ruthenium catalyst (5 mol %) and degassed with argon. Toluene (0.50 mL) was then added, followed by the addition of 1-(furan-2-yl)-*N*-(2-(piperidin-1-yl)ethyl)methanimine (**1**, 25.25 mg, 0.123 mmol, 1 equiv) and vinyltriethoxysilane (70.40 mg, 0.370 mmol, 3 equiv). 105 µL of the reaction mixture were loaded into the injector loop to promote the flow chemistry reaction. The final products are completely recovered (detected by UV), and the solvent is evaporated under reduced pressure. *p*-Dinitrobenzene is added to the residue and the mixture is dissolved in $CDCl_3$ and analyzed by ^1H NMR.

Note: In order to avoid using too much reagent during the different optimization processes, the whole reaction mixture in the HPLC injector will be pushed by the solvent and recovered after a certain time.

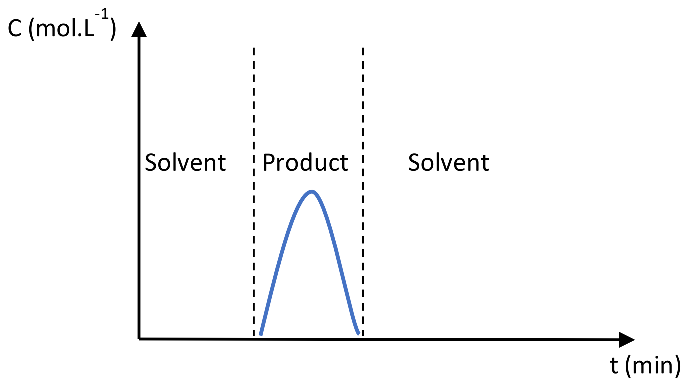
C (mol.L-1)
Solvent
Product
Solvent
t (min)

Table S1: Optimization of the catalyst for the alkylation reaction on the home-made setup : GP3 with one reactor.

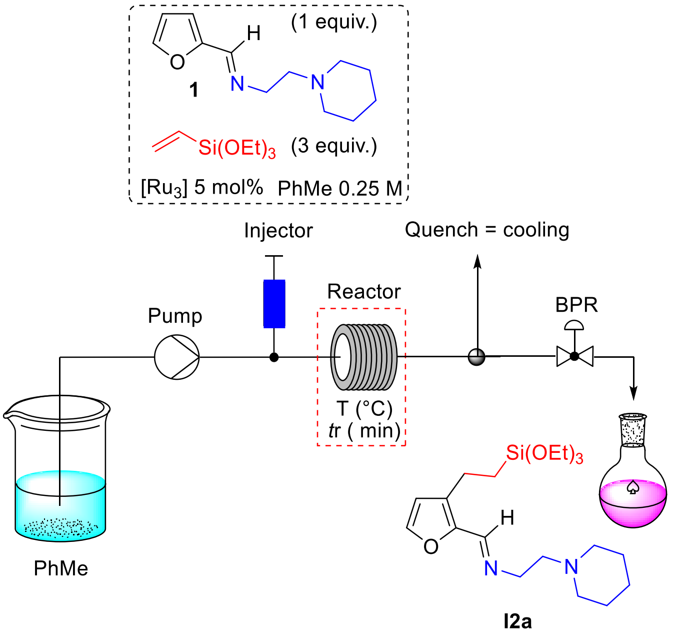

Entry	[Ru₃]	*tr* (min)	*T* (°C)	*p* (bar)	Conv. (%)[a]	Yield (%)[a]
**1**	$Ru_3(CO)_{12}$	90	165	130	58	45
**2**	$Ru_3(CO)_{12}$	152	165	130	84	58
**3**	$Ru_3(CO)_{12}$	6	200	127	39	36
**4**	$Ru_3(CO)_{12}$	9	200	133	40	30
**5**	$Ru_3(CO)_{12}$	6	250	138	83	63[b]
**6**	$Ru_3(CO)_{12}$	10	250	138	80	57
**7**	$Ru_3(CO)_{12}$	30	200	126	96	65
**8**	**comp1**	15	200	139	51	47
**9**	**comp1**	15	200	123	66	56
**10**	**comp1**	15	200	135	79	63
**11**	**comp1**	15	200	130-135	95	55

a: Conversions and yields are calculated by ^{1}H NMR using *p*-dinitrobenzene as internal standard. b: non-repeatable result, repeated four times with four different results from 75% to 53%.

Table S2: Optimization of the catalyst for the alkylation reaction on the home-made setup : GP3 with two reactors.

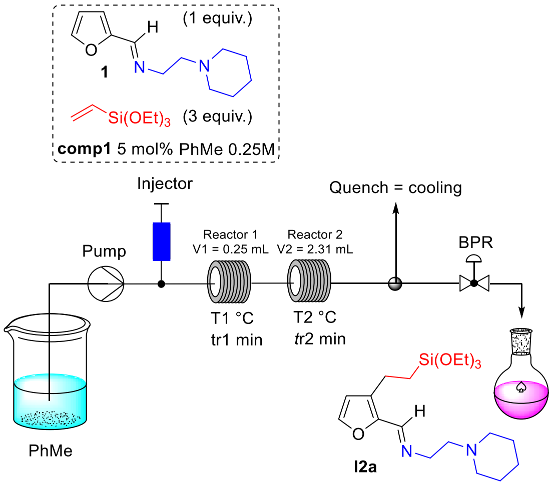

Entry	*tr* 1 (min)	*tr* 2 (min)	*T1* (°C)	*T2* (°C)	Conv. (%)[a]	yield (%)[a]
**1**	5	46	80	200	83	53
**2**	5	46	100	200	96	61
**3**	3,24	30	100	200	69	45
**4**	5	46	100	200	79	63
**5**	5	46	110	200	85	62
**6**	5	46	130	200	87	70
**6**	5	46	150	200	88	70
**7**	5	46	130	140	39	38
**8**	5	46	130	160	-	71
**9**	5	46	130	180	-	79

a : Yields and conversions calculated by ^{1}H NMR using *p*-dinitrobenzene as internal standard.

P ≈ 130 bar

2. Second optimization with a continuous flow chemistry system (Vapourtec)

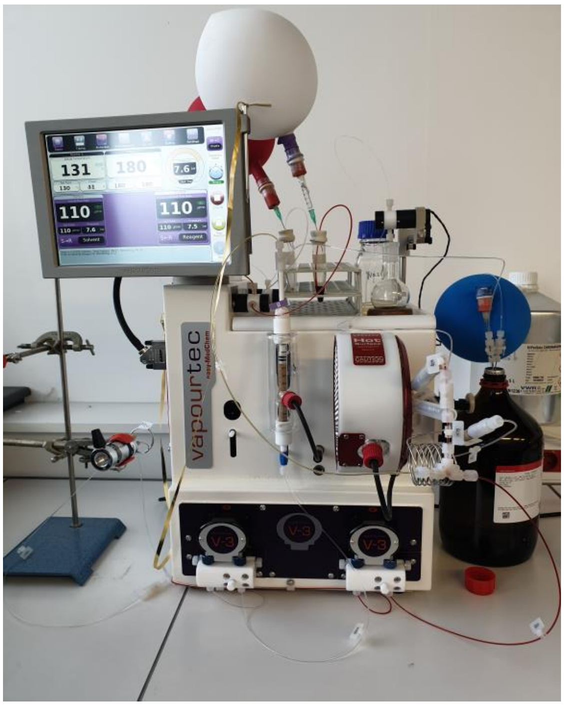

A Vapourtec E-Series assembly comprising:

- a mixture A containing furfural (0.7 M) and 2-(piperidin-1-yl)ethane-1,2-diamine (0.7 M), which is passed through a fixed bed reactor 1 filled with magnesium sulfate
- a mixture B containing vinyltriethoxysilane (1.05 or 2.1 M) and a ruthenium catalyst (1 to 5 mol % with respect to furfural), which is introduced at the outlet of the fixed bed reactor
- The flow rates of pumps A and B are equal
- 1 mL stainless steel coil immersed in an oil bath at 130 °C is used as reactor 2, and is installed before the 10 mL reactor 3

General procedure 4 (GP4):

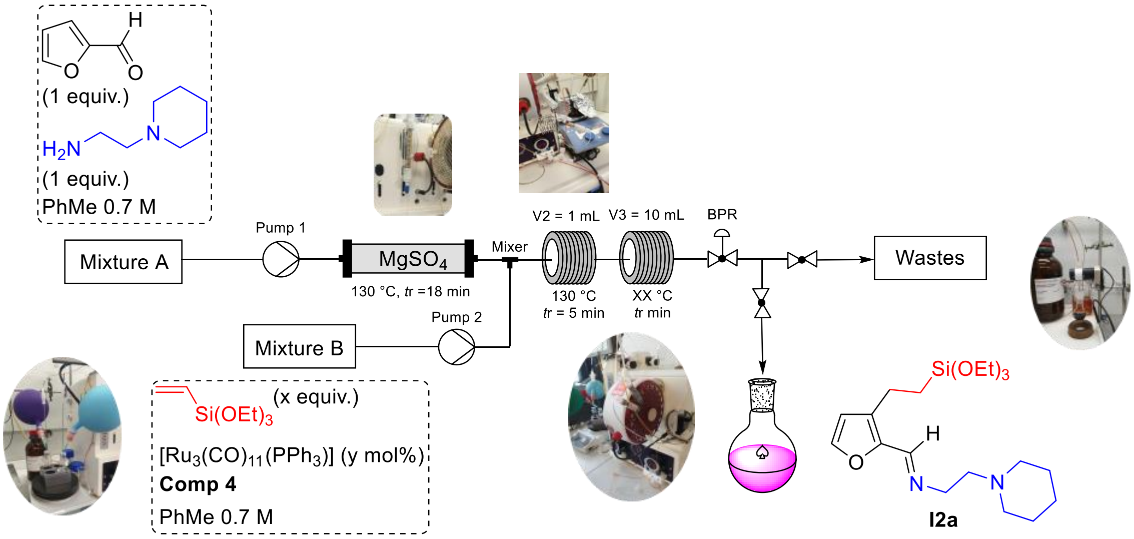

Mixture A: An oven-dried sealed tube equipped with a magnetic stirrer under argon, was loaded with furfural (240.20 mg, 2.50 mmol, 1 equiv), 2-(pyperidin-1-yl)ethanamine (320.55 mg, 2.50 mmol, 1 equiv) and filled with dried toluene to a total volume of 3.5 mL.

Mixture B: An oven-dried sealed tube equipped with a magnetic stirrer, was loaded with triphenylphosphine triruthenium undecacarbonyl (y mol % with regards to furfural) and degassed with argon. Vinyltriethoxysilane (x equiv with regards to furfural) was then added to the middle, and the mixture was filled with dried toluene to a total volume of 3.5 mL. The mixture was stirred at room temperature to completely dissolve the catalyst.

Pumps 1 and 2 were operated at the same flow rate.

The reaction was then monitored by continuous flow. The solution A is pumped into pump 1 and passed through the packed bed reactor which is at 130 °C containing $MgSO_4$. The residence time depends on the intrinsic volume (*V*i) of this reactor, and is kept constant at ≈ 18 min.

Determining the intrinsic volume

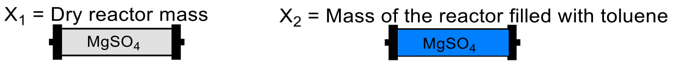

$$Vi = \frac{X2 - X1}{\rho_{toluene}}$$

$\rho_{toluene}$ = Volumic mass of toluene

The solution B is pumped through pump B. The mixture of the two solutions A and B passed first through the coil reactor at 130 °C and then into a second coil reactor at the desired temperature. Product recovery is initiated when the system reaches a steady state, based on the dispersion curves given by the apparatus. After reaching the steady state an aliquot of the product was taken for ^{1}H NMR analysis using *p*-dinitrobenzene as an internal standard. It should be noted that this internal standard is inert to the reaction components, and that no degradation of it was observed.

Scatter plot; concentration versus time. The stationary state is at the plateau when the concentration does not vary anymore.

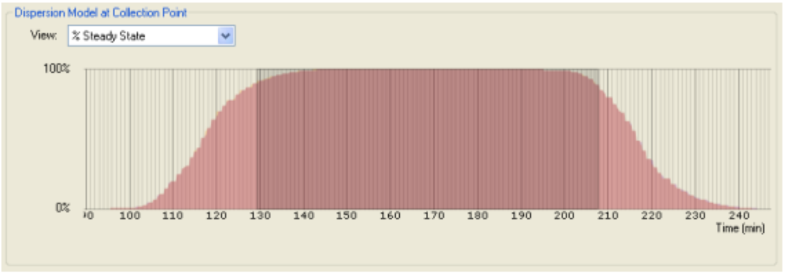

Dispersion prediction in Vapourtec Flow Commander ™ software

V. Scope of the C3-alkylation of furfural in continuous flow

3-(2-(Triethoxysilyl)ethyl)furan-2-carbaldehyde (2a)

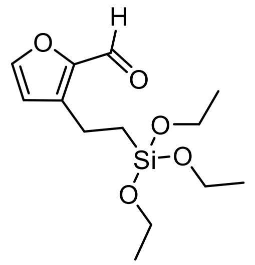

Chemical Formula: $C_{13}H_{22}O_5Si$
Molecular Weight: 286,39900

Prepared according to **GP4**: the reaction of mixture A containing, furfural (240.20 mg, 2.50 mmol, 0.7 M) and 2-(piperidin-1-yl)ethanamine (320.55 mg, 2.50 mmol, 0.7 M), with mixture B containing triruthenium undecacarbonyl (22 mg, 0.025mmol, 0.007 M) and vinyltriethoxysilane (1.43 g, 7.50 mmol, 1.07 M) was conducted by continuous flow chemistry , residence time 1 = 18 min, residence time 2 = 50 min. An aliquot of 0.5 mL of the product mixture was evaporated (93% conv., 77% NMR yield), and the crude was purified by silica gel column chromatography eluting with a mixture of cyclohexane/EtOAc 9:1 to give 38 mg of the desired product as an orange oil (75% yield). **^{1}H NMR** (300 MHz, $CDCl_3$) δ 9.76 (s, 1H), 7.54 (d, *J* = 1.7 Hz, 1H), 6.49 (d, *J* = 1.7 Hz, 1H), 3.81 (q, *J* = 7.0 Hz, 6H), 2.95 – 2.84 (m, 2H), 1.22 (t, *J* = 7.0 Hz, 9H), 1.01 – 0.90 (m, 2H). These data are in good agreement with those reported in literature.[2]

3-(2-(Triisopropoxysilyl)ethyl)furan-2-carbaldehyde (2b)

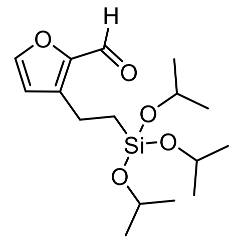

Chemical Formula: $C_{16}H_{28}O_5Si$
Molecular Weight: 328,48000

Prepared according to **GP4**: the reaction of mixture A containing, furfural (240.20 mg, 2.50 mmol, 0.7 M) and 2-(piperidin-1-yl)ethanamine (320.55 mg, 2.50 mmol, 0.7 M), with mixture B containing triruthenium undecacarbonyl (22 mg, 0.025mmol, 0.007 M) and vinyltriisopropoxysilane (1758 mg, 7.50 mmol, 1.07 M) was conducted by continuous flow chemistry residence time 1 = 18 min, residence time 2 = 50 min. An aliquot of 0.50 mL of the product mixture was evaporated (89% conv., 80% NMR yield), and the crude was purified by silica gel column chromatography eluting with a mixture of cyclohexane/EtOAc 9:1 to give 39 mg of the desired product as a yellow oil (67% yield). **^{1}H NMR** (400 MHz, $CDCl_3$) δ 9.76 (s, 1H), 7.54 (d, *J* = 1.7 Hz, 1H), 6.49 (d, *J* = 1.7 Hz, 1H), 4.22 (hept, *J* = 6.1 Hz, 3H), 2.93 – 2.84 (m, 2H), 1.19 (d, *J* = 5.9 Hz, 18H), 0.96 – 0.85 (m, 2H). **^{13}C NMR** (101 MHz, $CDCl_3$) δ 177.5, 147.8, 147.2, 141.3, 113.9, 65.1, 25.5, 18.47, 13.2. **HRMS (ESI)**: m/z: $[M+Na]^+$ Calculated for $C_{16}H_{28}O_5SiNa$ 351.1598. Found 351.1598. **IR**: ν (cm^{-1}) 2973, 2931, 2895, 2822, 2772, 2729, 2644, 1682, 1582, 1476, 1425, 1380, 1173, 1119, 1035, 890.

3-(2-(Triphenylsilyl)ethyl)furan-2-carbaldehyde (2c)

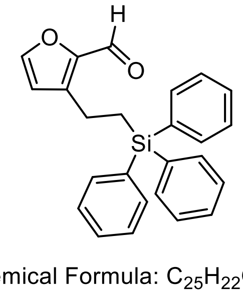

Chemical Formula: $C_{25}H_{22}O_2Si$
Molecular Weight: 382,53

Prepared according to **GP4**: the reaction of mixture A containing, furfural (240.20 mg, 2.50 mmol, 0.6 M) and 2-(piperidin-1-yl)ethanamine (320.55 mg, 2.50 mmol, 0.6 M), with mixture B containing triruthenium undecacarbonyl (22 mg, 0.025 mmol, 0.006 M) and triphenyl(vinyl)silane (2.14 g, 7.50 mmol, 1.83 M) was conducted by continuous flow chemistry residence time 1 = 18 min, residence time 2 = 50 min. An aliquot of 1 mL of the product mixture was evaporated (92% conv., 68% NMR yield), and the crude was purified by silica gel column chromatography eluting with a mixture

of cyclohexane/EtOAc 9:1 to give 80 mg of the desired product as a white solid (70% yield). **^{1}H NMR** (400 MHz, $CDCl_3$) δ 9.63 (s, 1H), 7.64 – 7.54 (m, 6H), 7.52 (d, *J* = 1.7 Hz, 1H), 7.48 – 7.35 (m, 9H), 6.45 (d, *J* = 1.7 Hz, 1H), 2.99 – 2.90 (m, 2H), 1.76 – 1.66 (m, 2H). These data are in good agreement with those reported in literature.[2]

3-(2-(Triethylsilyl)ethyl)furan-2-carbaldehyde (2d)

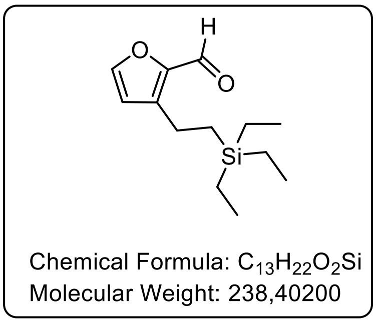

Prepared according to **GP4**: the reaction of mixture A containing, furfural (240.20 mg, 2.50 mmol, 0.7 M) and 2-(piperidin-1-yl)ethanamine (320.55 mg, 2.50 mmol, 0.7 M), with mixture B containing triruthenium undecacarbonyl (22 mg, 0.025mmol, 0.007 M) and vinyltriethylsilane (1.07 g, 7.50 mmol, 1.07 M) was conducted by continuous flow chemistry residence time 1 = 18 min, residence time 2 = 50 min. An aliquot of 0.80 mL of the product mixture was evaporated (51% conv., 46% NMR yield), and the crude was purified by silica gel column chromatography eluting with a mixture of cyclohexane/EtOAc 9:1 to give 27 mg of the desired product as a yellow oil (40% yield). **^{1}H NMR** (300 MHz, $CDCl_3$) δ 9.76 (s, 1H), 7.55 (d, *J* = 1.7 Hz, 1H), 6.49 (d, *J* = 1.7 Hz, 1H), 2.85 – 2.73 (m, 2H), 0.96 (t, *J* = 7.9 Hz, 9H), 0.90 – 0.81 (m, 2H), 0.57 (qd, *J* = 7.9, 0.8 Hz, 6H). **^{13}C NMR** (101 MHz, $CDCl_3$) δ 177.75, 147.69, 147.28, 141.67, 113.84, 19.19, 12.61, 7.39, 3.16. **HRMS (ESI)**: m/z $[M+H]^+$ Calculated for $C_{13}H_{22}O_2SiH$ 239.1462. Found 239.1460. **IR**: ν (cm^{-1}) 2953, 2910, 2875, 2819, 2779, 2732, 1680, 1581, 1475, 1375, 1016, 879.

3-((2*R,4*R**)-bicyclo[2.2.1]heptan-2-yl)furan-2-carbaldehyde (2e)**

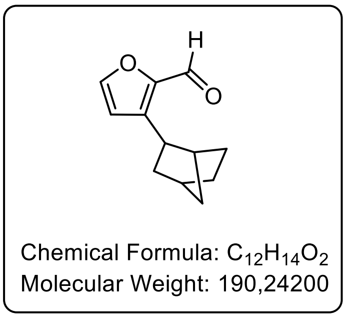

Prepared according to **GP4**: the reaction of mixture A containing, furfural (240.20 mg, 2.50 mmol, 0.7 M) and 2-(piperidin-1-yl)ethanamine (320.55 mg, 2.50 mmol, 0.7 M), with mixture B containing triruthenium undecacarbonyl (22 mg, 0.025 mmol, 0.007 M) and norbornene (706.20 mg, 7.50 mmol, 1.07 M) was conducted by continuous flow chemistry residence time 1 = 18 min residence time 2 = 50 min. An aliquot of 1.00 mL of the product mixture was evaporated (58% conv., 47% NMR yield), and the crude was purified by silica gel column chromatography eluting with a mixture of cyclohexane/EtOAc 95:5 to give 33 mg of the desired product as a yellow oil (49% yield). **^{1}H NMR** (300 MHz, $CDCl_3$) δ 9.78 (s, 1H), 7.53 (d, *J* = 1.7 Hz, 1H), 6.50 (d, *J* = 1.7 Hz, 1H), 3.18 (dd, *J* = 9.0, 5.4 Hz, 1H), 2.36 (d, *J* = 4.8 Hz, 1H), 2.27 (d, *J* = 3.3 Hz, 1H), 1.92 – 1.78 (m, 1H), 1.68 – 1.45 (m, 4H), 1.45 – 1.33 (m, 1H), 1.32 – 1.22 (m, 2H). **^{13}C NMR** (101 MHz, $CDCl_3$) δ 177.84, 147.75, 147.21, 144.33, 112.06, 43.51, 39.60, 37.24, 36.69, 36.60, 30.16, 28.48. **HRMS (ESI)**: m/z $[M+H]^+$ Calculated for $C_{12}H_{14}O_2H$ 191.1067. Found 191.1065. **IR**: ν (cm^{-1}) 2953, 2931, 2871, 2821, 2780, 2728, 1674, 1574, 1472, 1456, 1425, 1374, 1344, 1298.

VI. Copies of NMR spectra

(Diphenyl(2-(triethoxysilyl)ethyl)phosphine)triruthenium undecacarbonyl (Comp1)

^{1}H

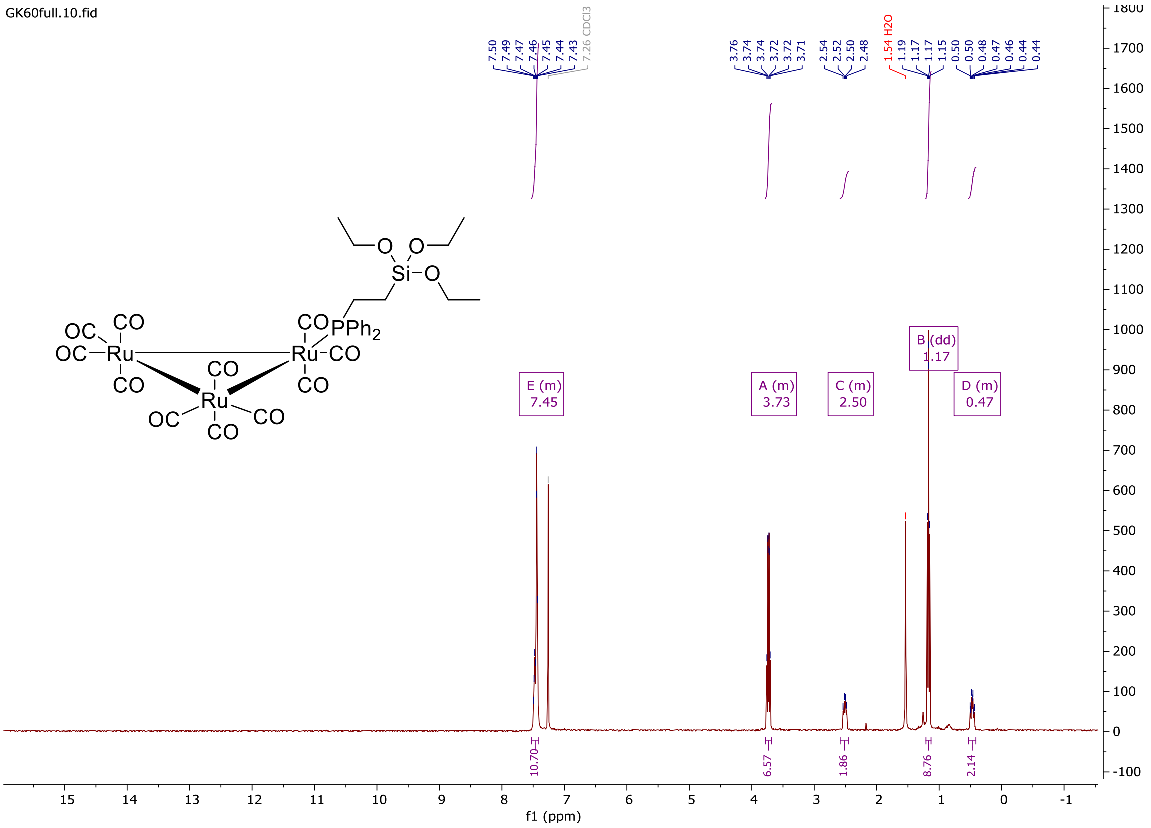

^{31}P

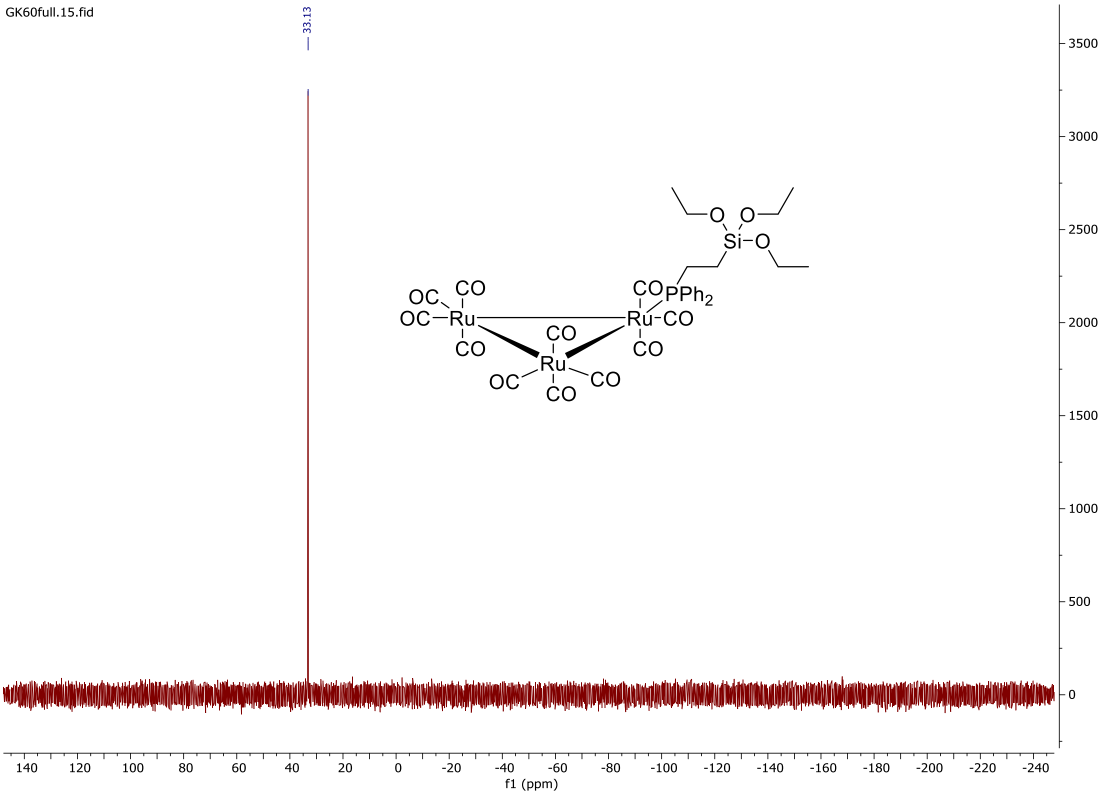

Bis(diphenyl(2-(triethoxysilyl)ethyl)phosphine)triruthenium decacarbonyl (comp2)

^{1}H

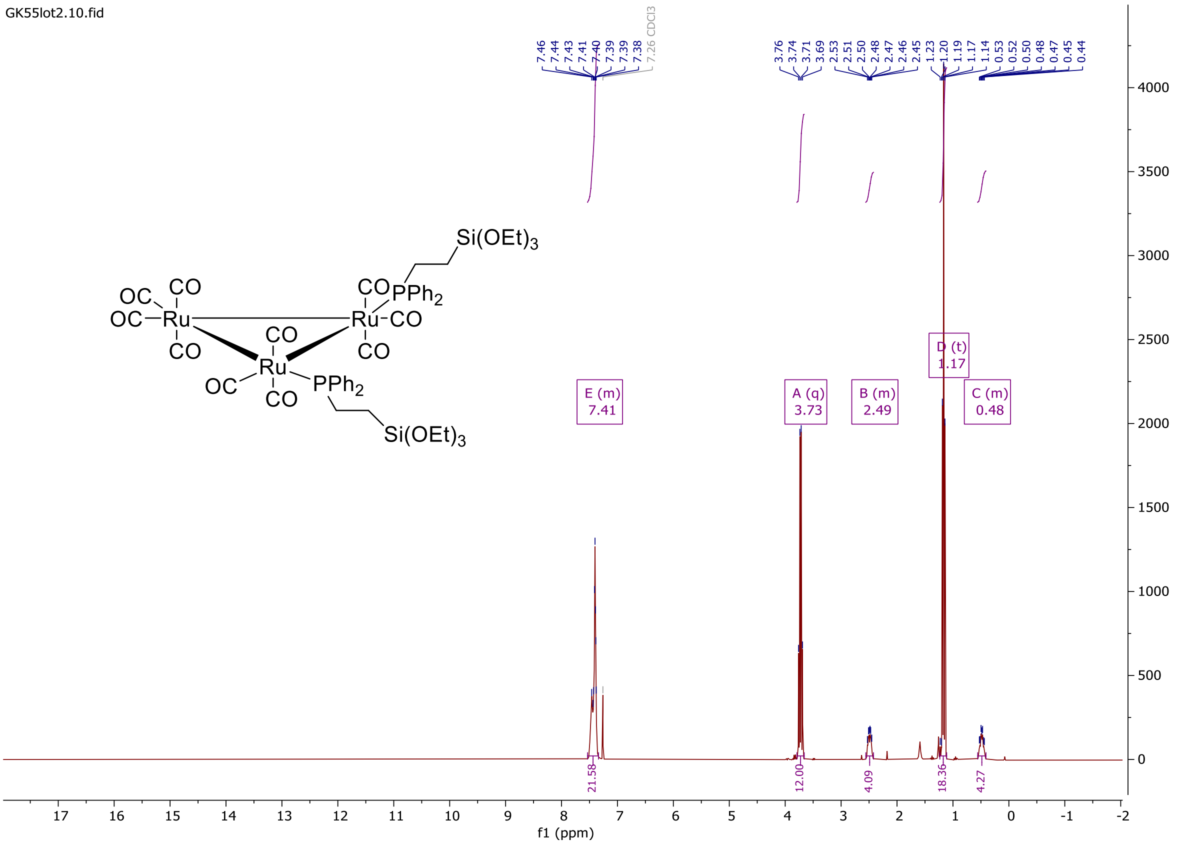

^{31}P

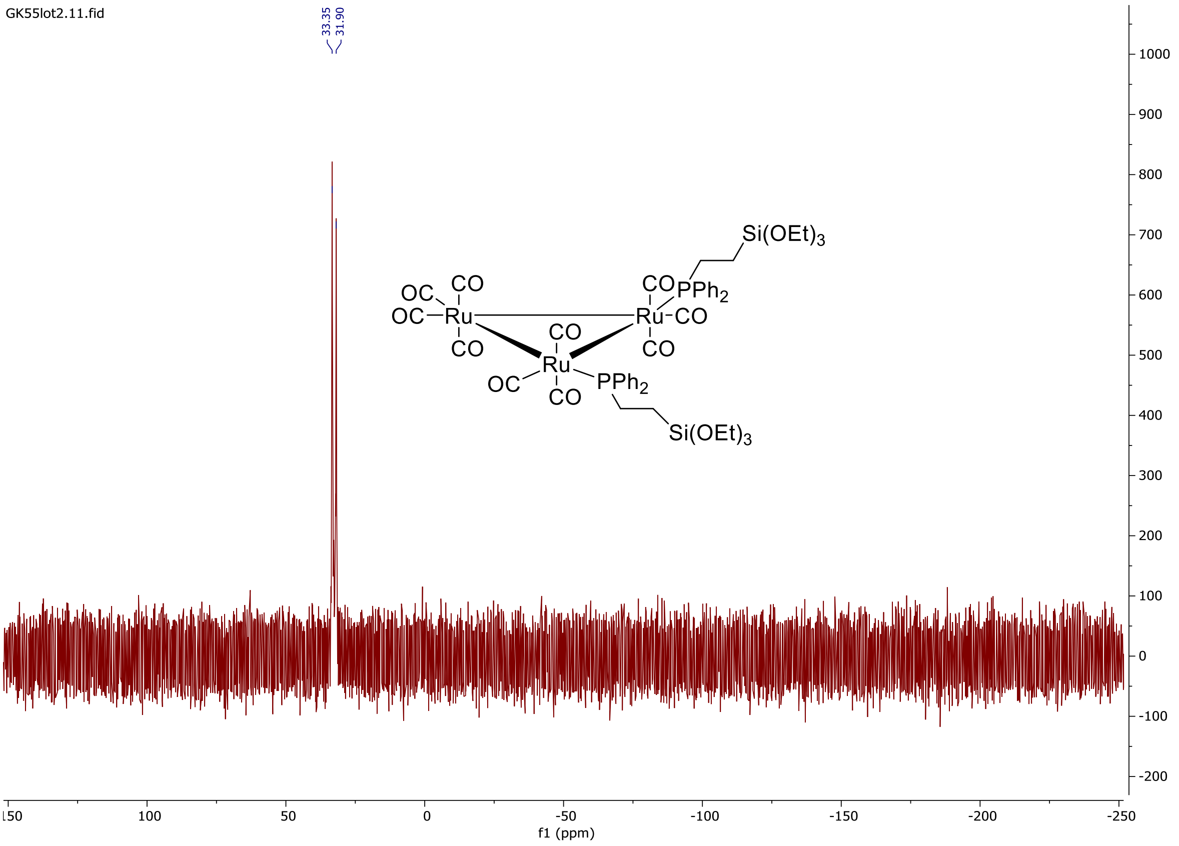

Tris(diphenyl(2-(triethoxysilyl)ethyl)phosphane)triruthenium nonacarbonyl (comp3)

^{1}H

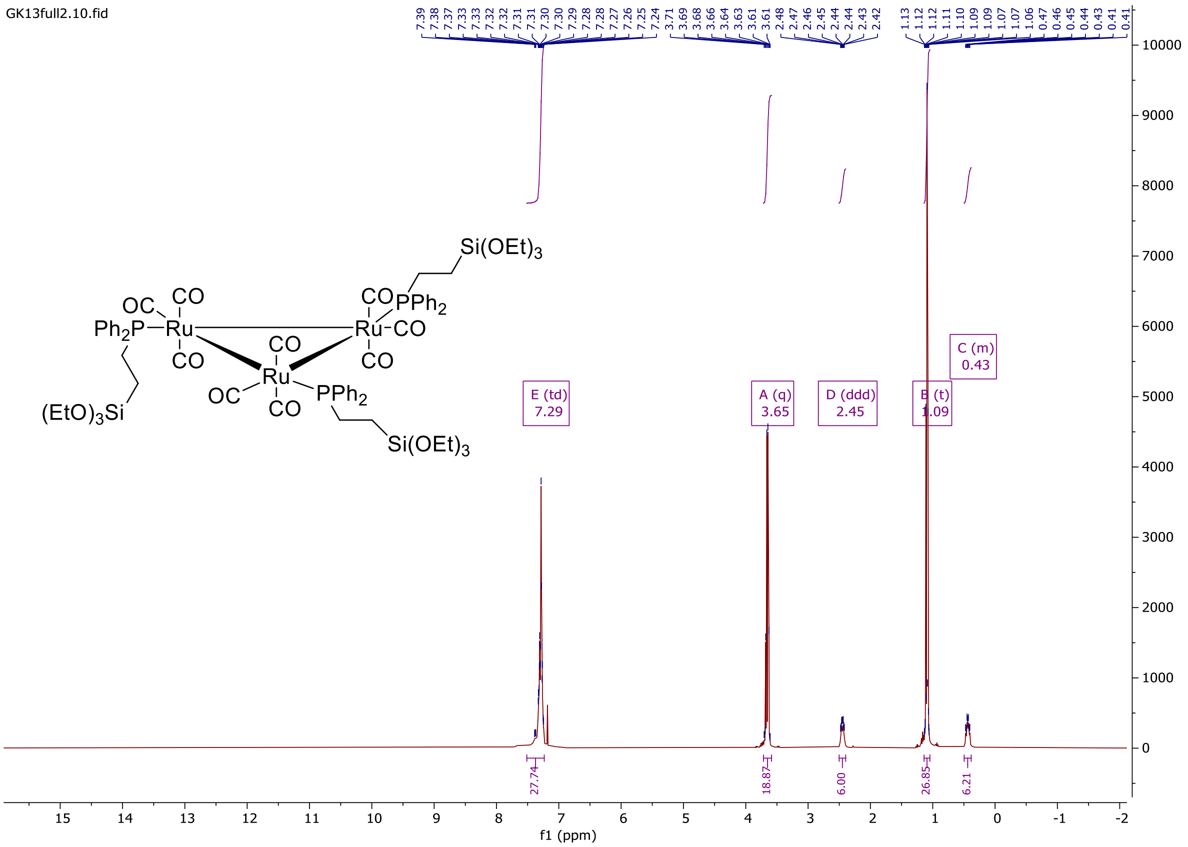

^{13}C

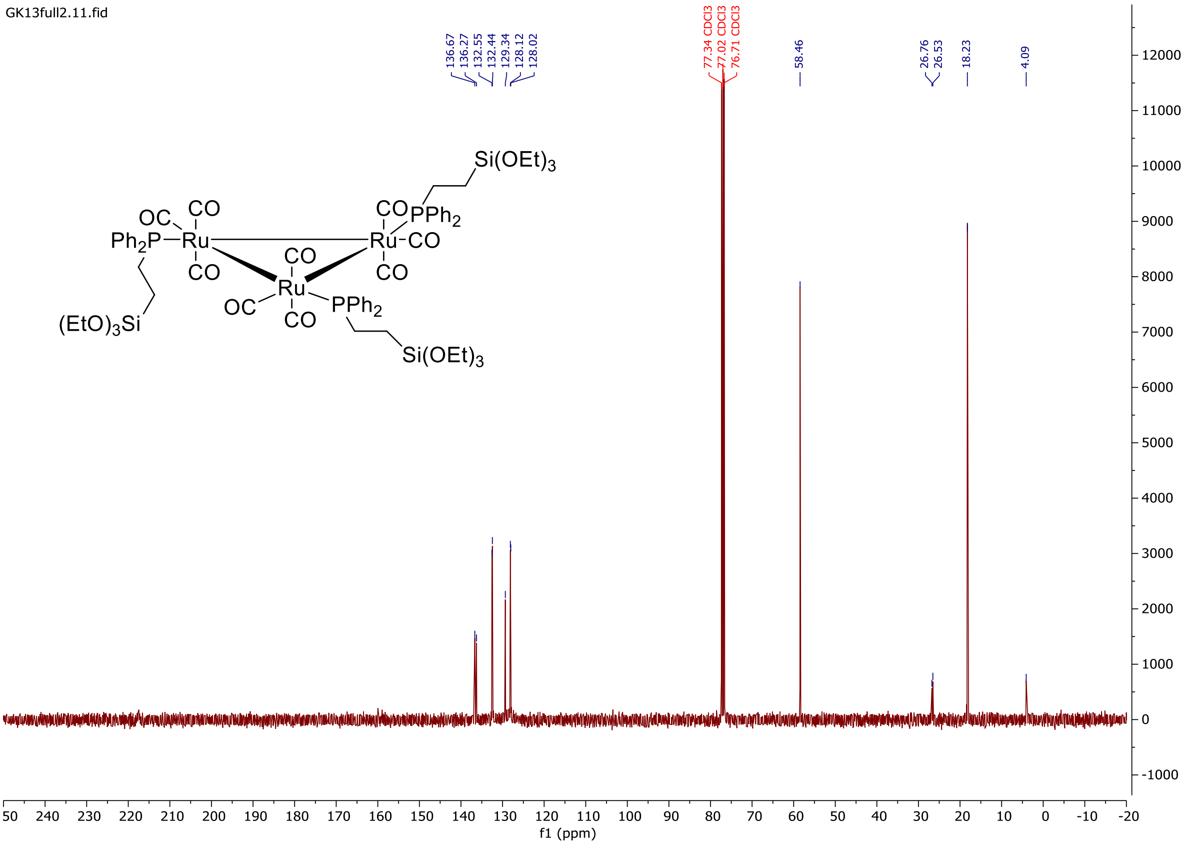

^{31}P

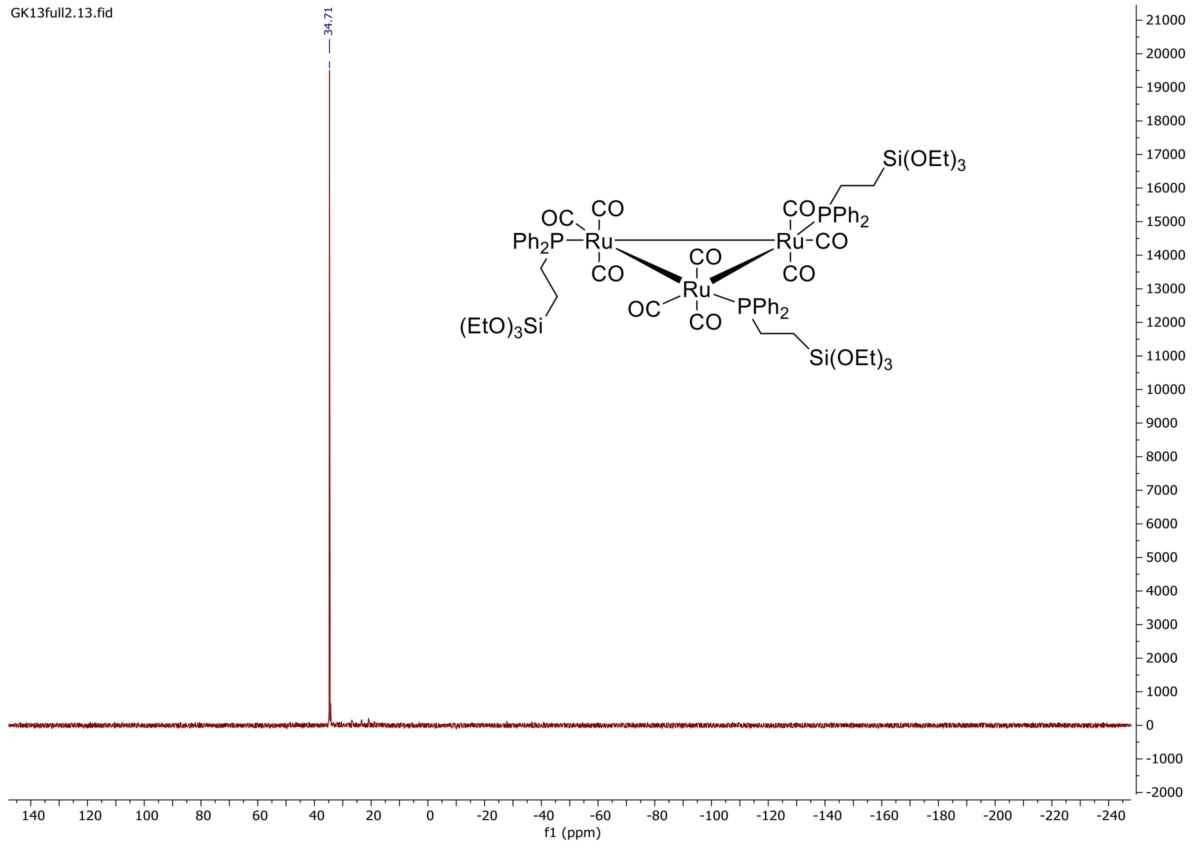
GK13full2.13.fid
34.71
Si(OEt)3
CO
PPh2
OC
CO
Ph2P
Ru
Ru
CO
CO
CO
CO
Ru
OC
PPh2
CO
(EtO)3Si
Si(OEt)3
f1 (ppm)

Triphenylphosphine triruthenium undecacarbonyl (comp4)

^{1}H

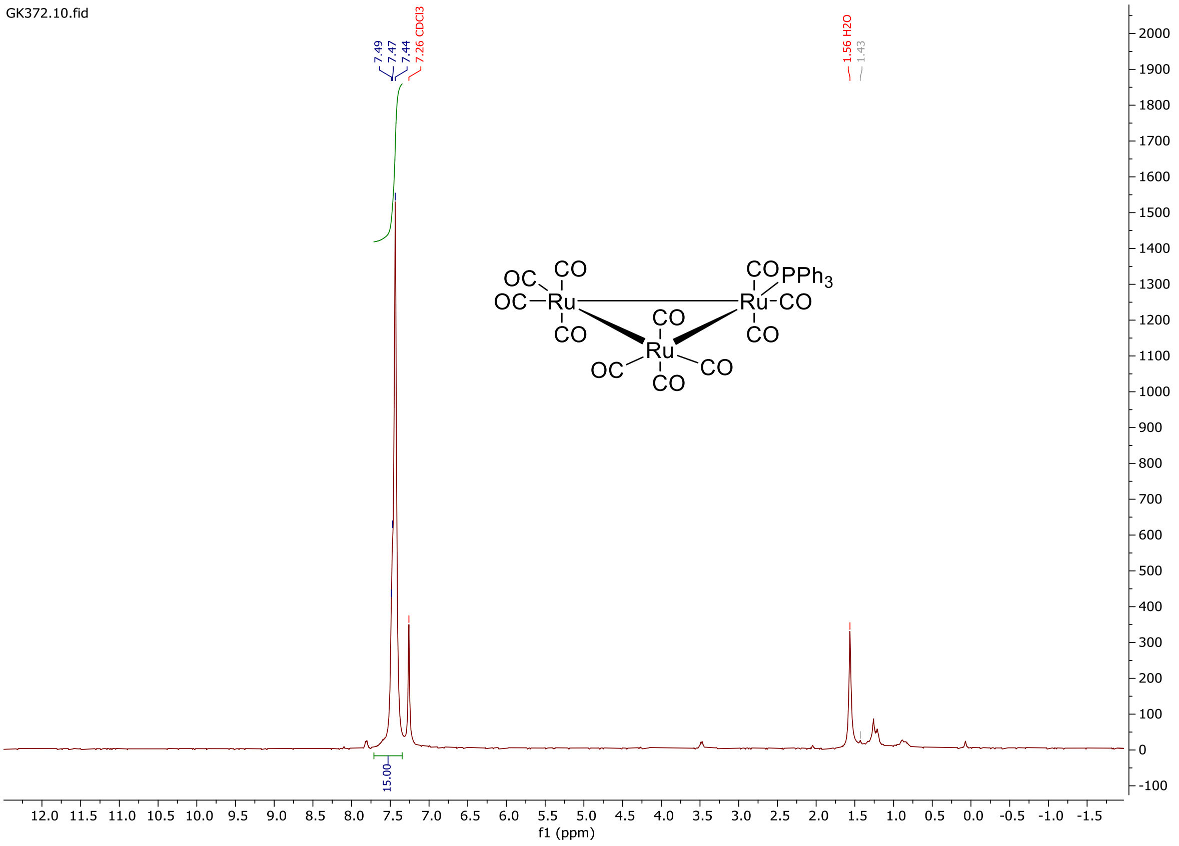

^{31}P

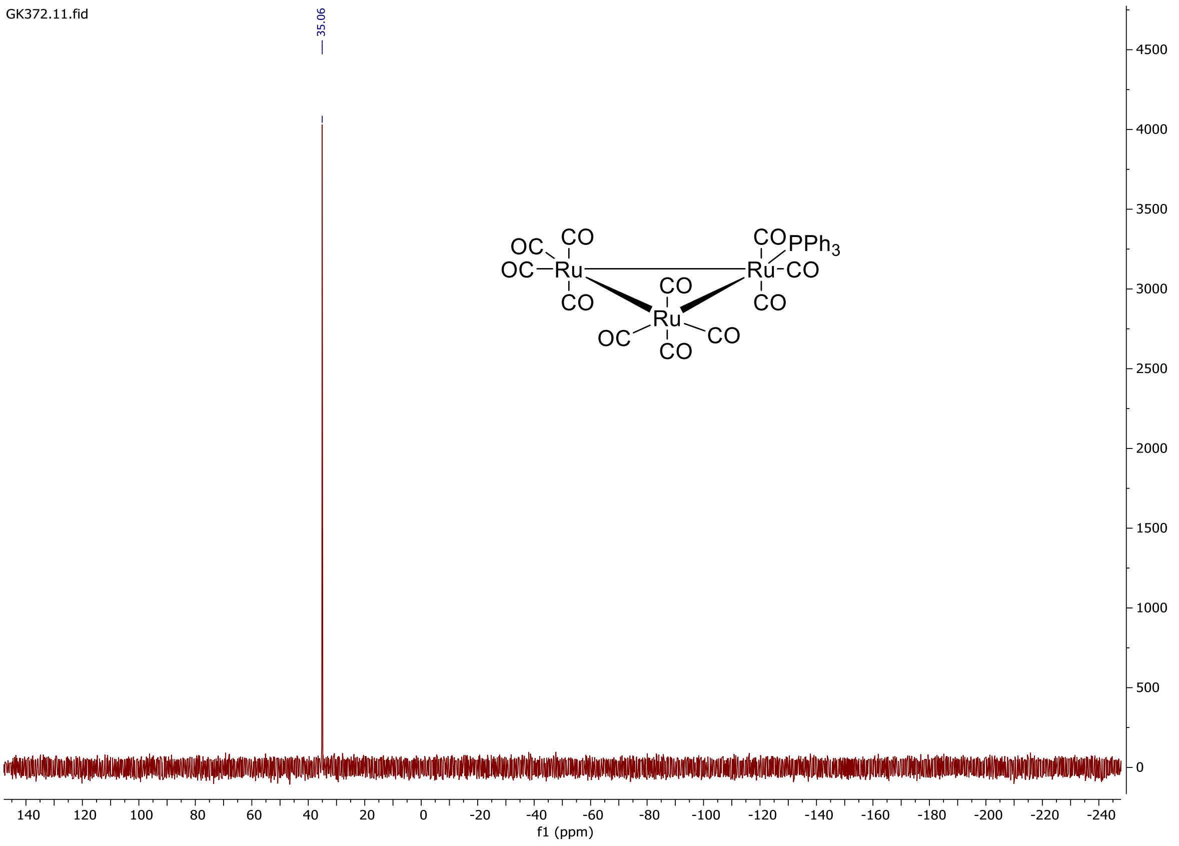

3-(2-(Triisopropoxysilyl)ethyl)furan-2-carbaldehyde (2b)

[1]H

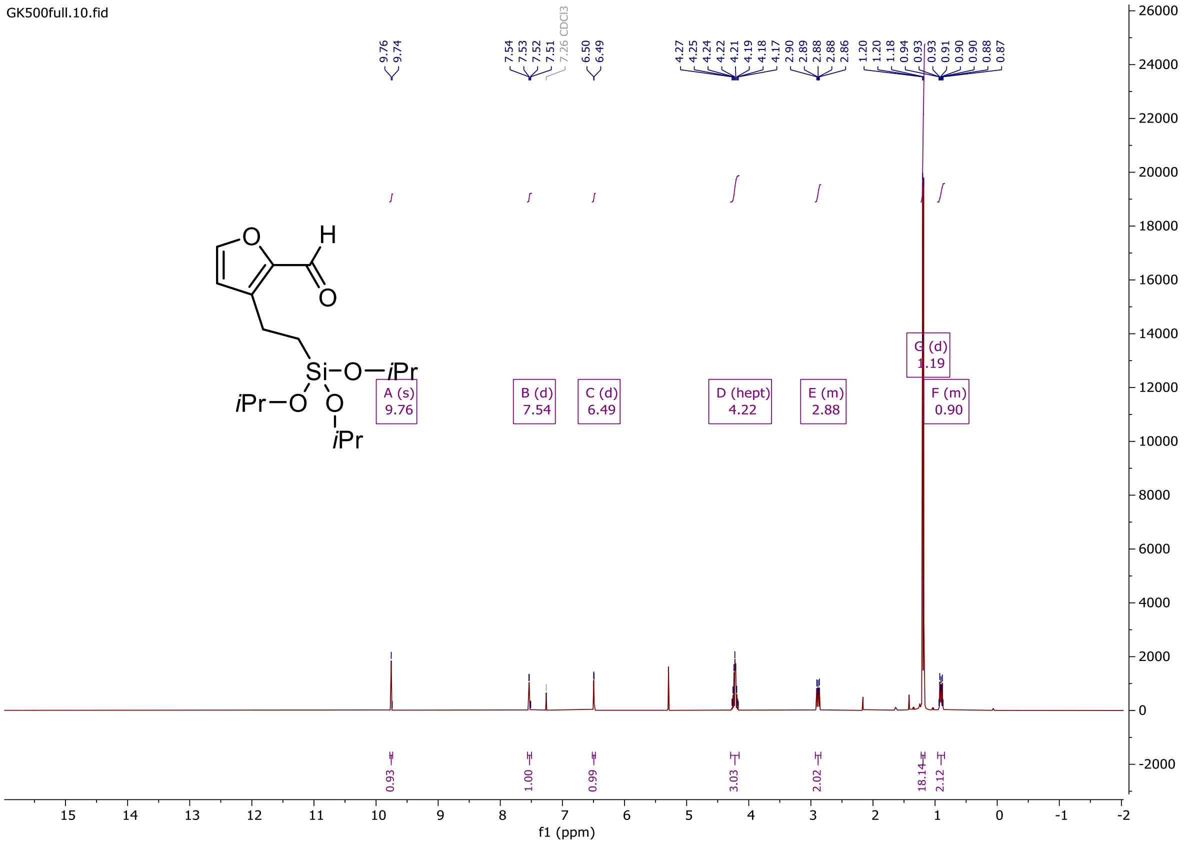

[13]C

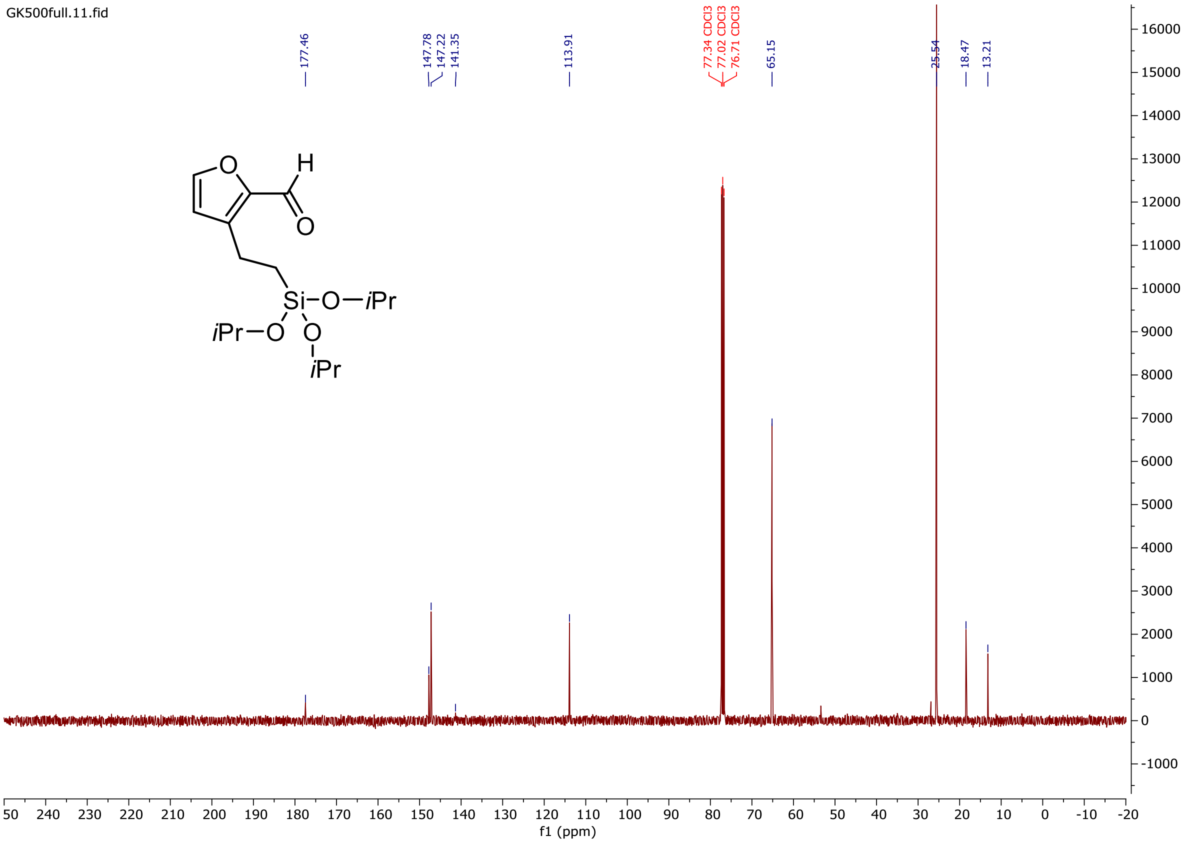

3-(2-(Triethylsilyl)ethyl)furan-2-carbaldehyde (2d)

^{1}H

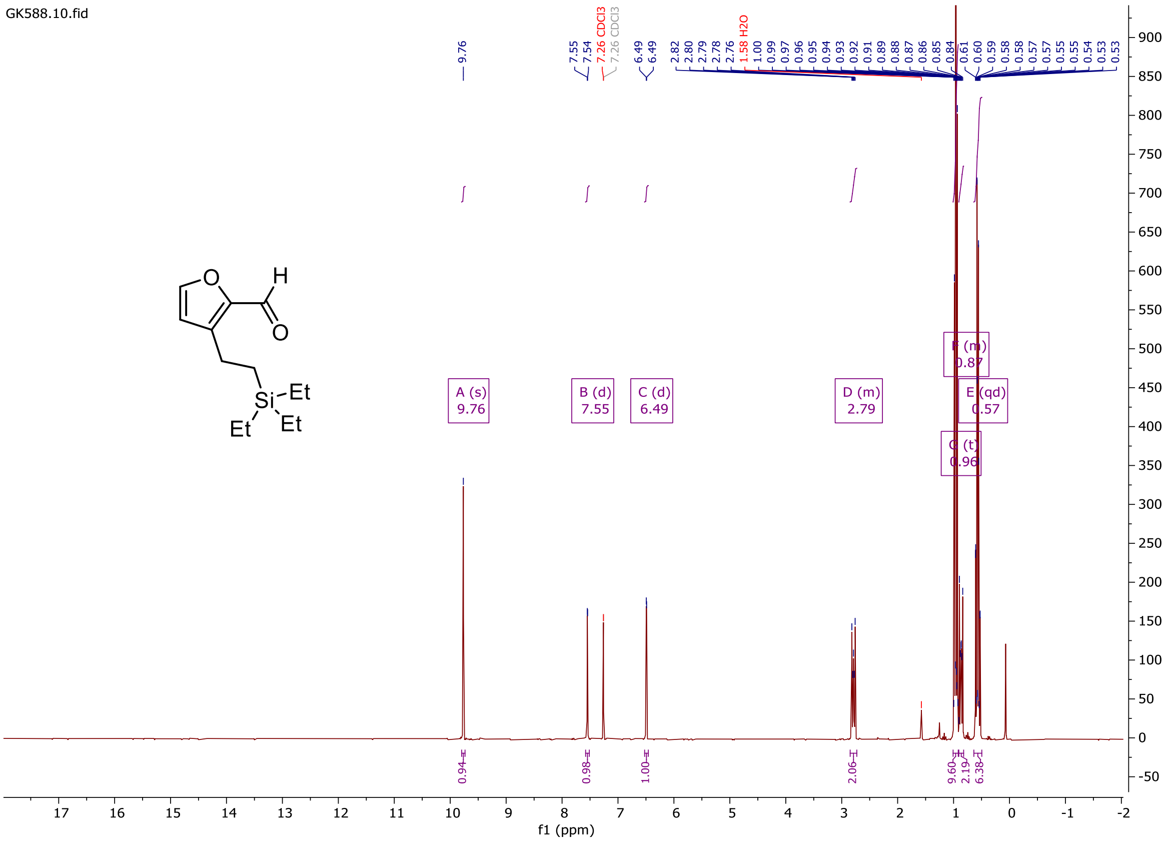

^{13}C

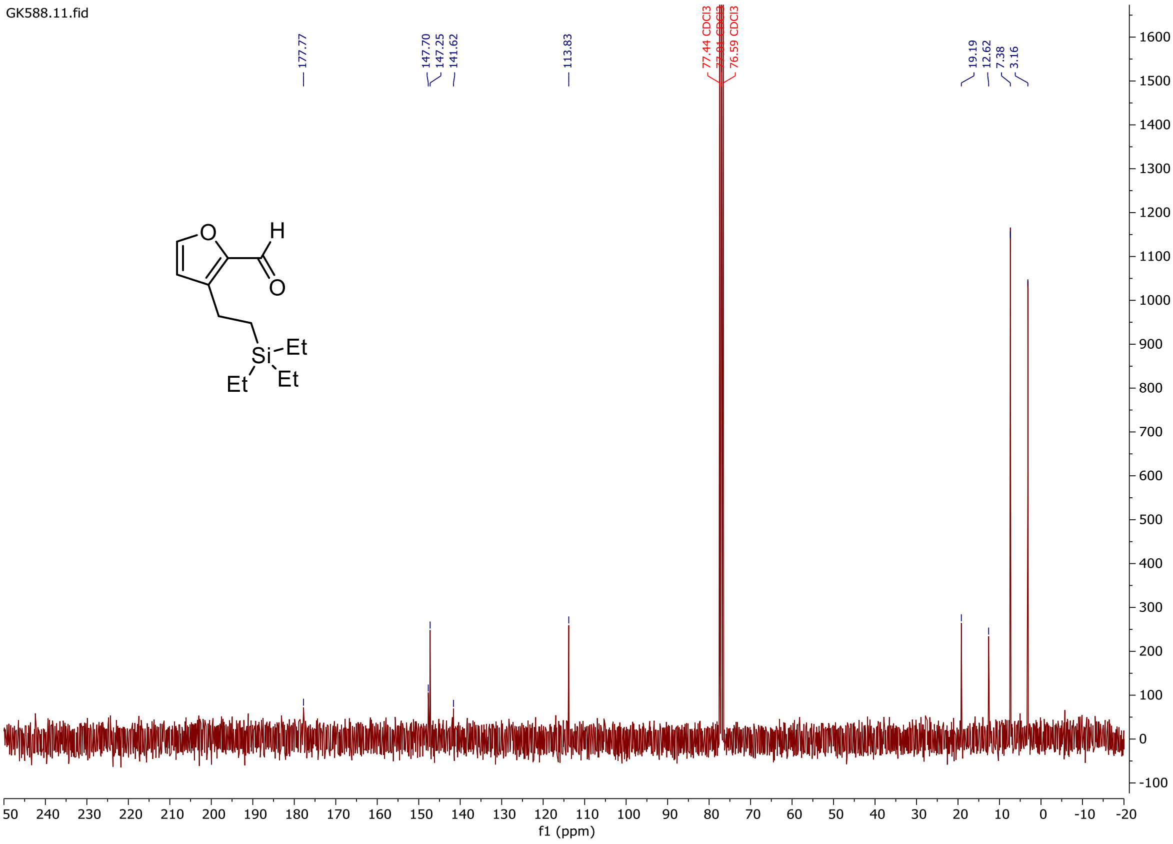

3-((2*R**,4*R**)-Bicyclo[2.2.1]heptan-2-yl)furan-2-carbaldehyde (2e)

^{1}H

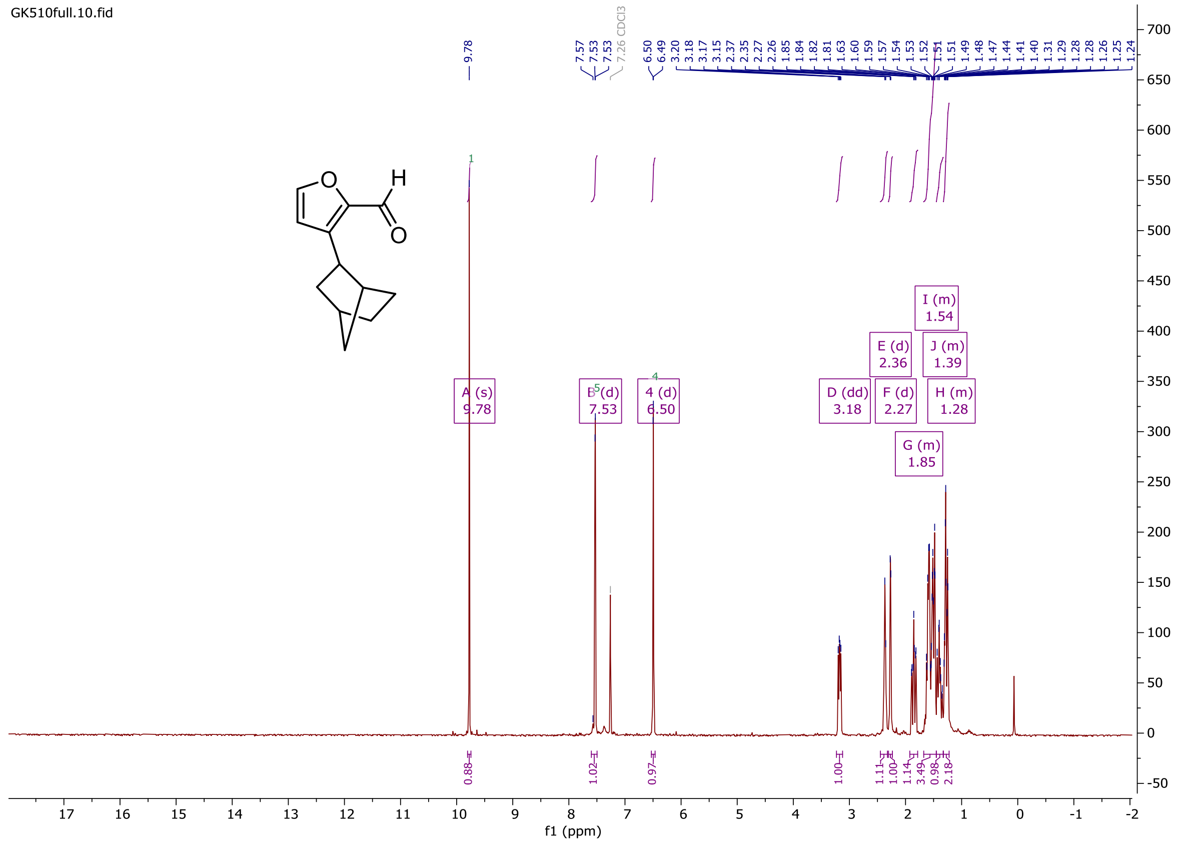

^{13}C

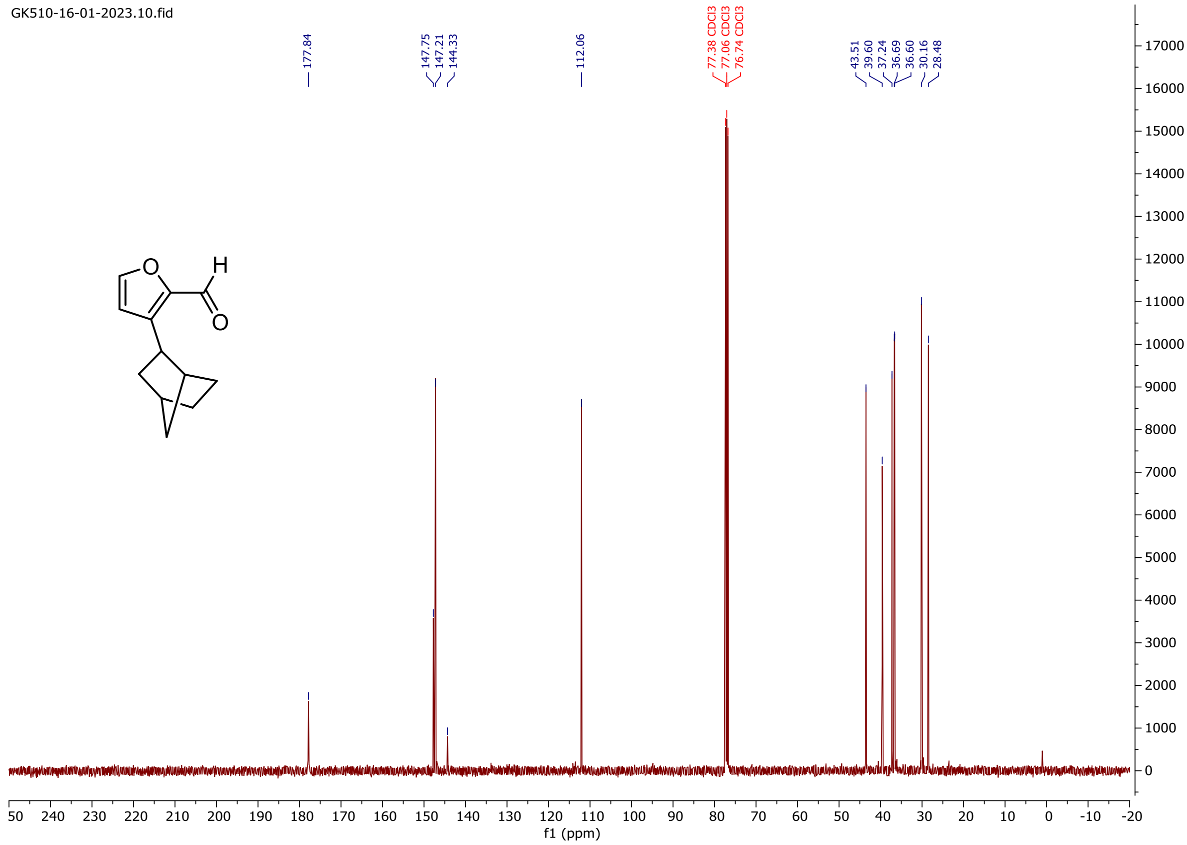

Spectrum of ruthenium aggregates in CD_2Cl_2

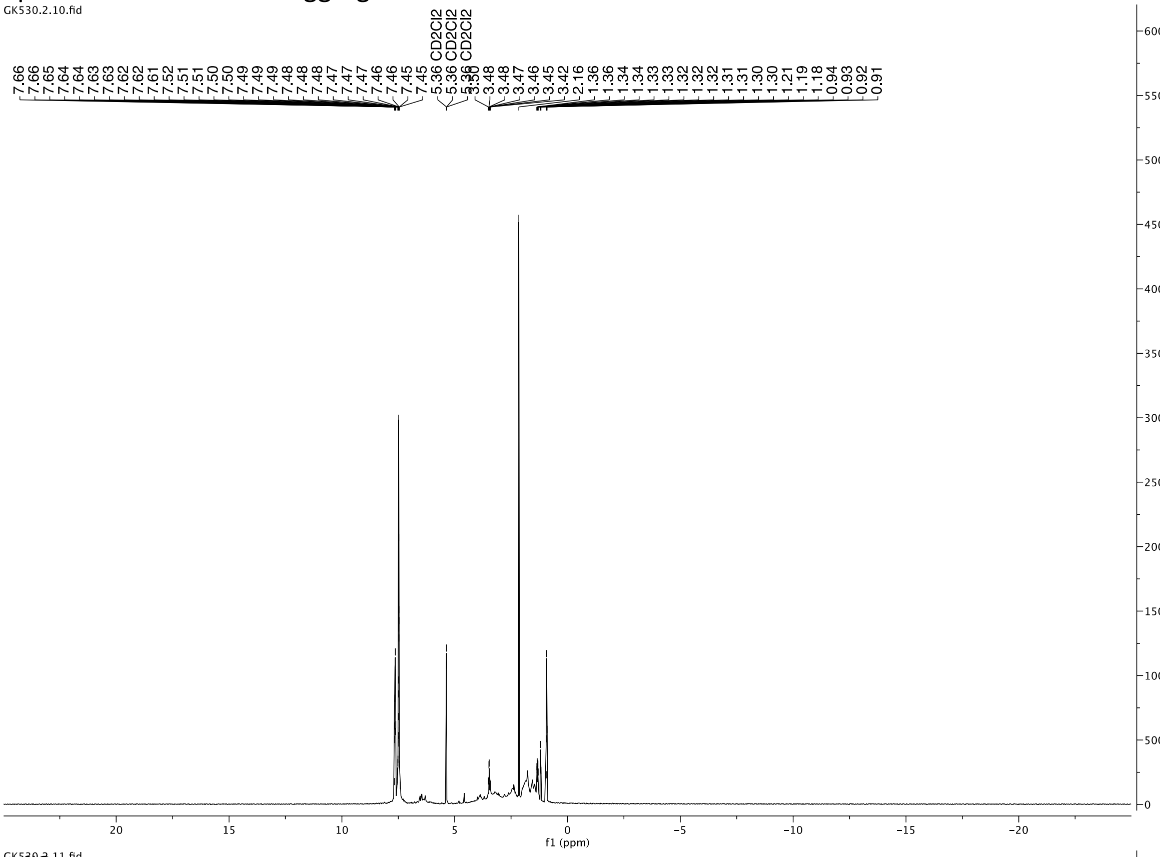

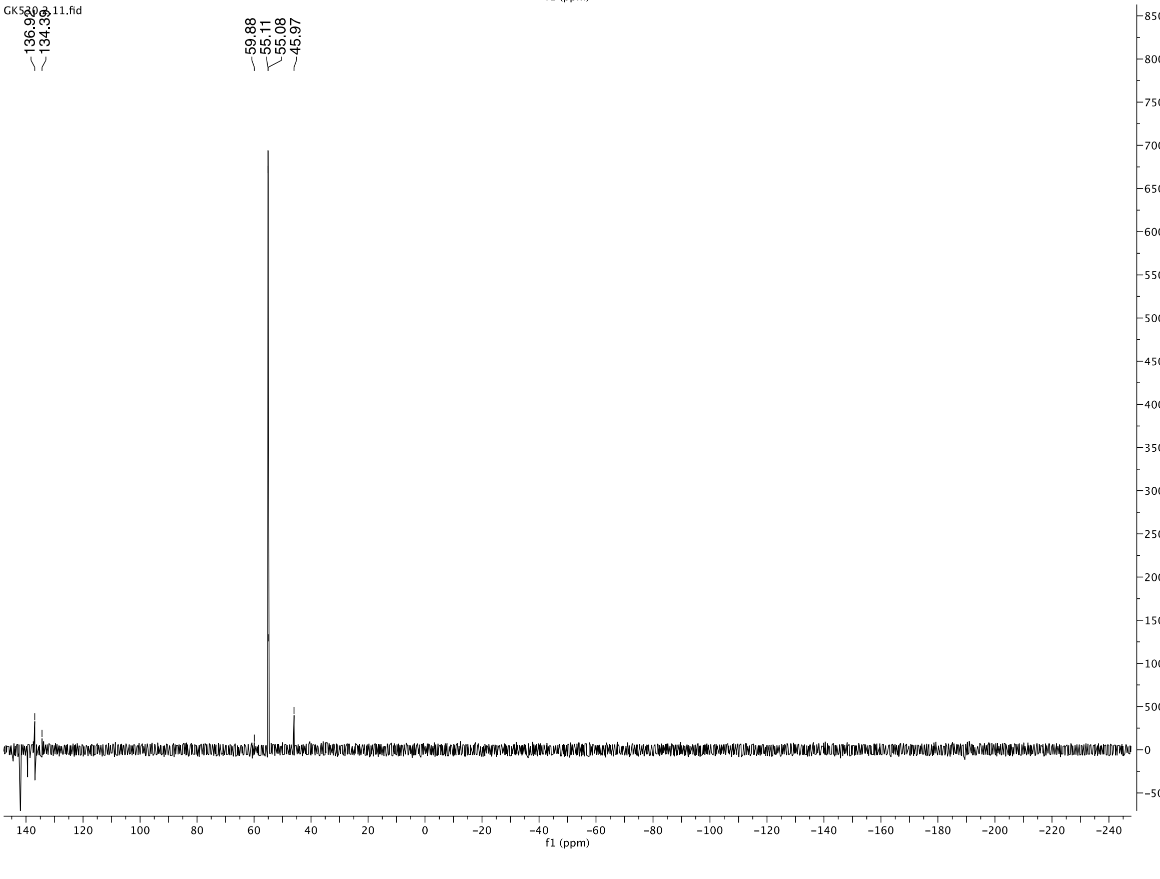

Supplement: File 1 — Experimental and copies of spectra. [file Beilstein_J_Org_Chem-19-582-s001.pdf]
